# Effect of Drug Loading Method and Drug Physicochemical Properties on the Material and Drug Release Properties of Poly (Ethylene Oxide) Hydrogels for Transdermal Delivery

**DOI:** 10.3390/polym9070286

**Published:** 2017-07-19

**Authors:** Rachel Shet Hui Wong, Kalliopi Dodou

**Affiliations:** School of Pharmacy and Pharmaceutical Sciences, Faculty of Health Sciences and Wellbeing, University of Sunderland, Science Complex, Wharncliffe Street, Sunderland SR1 3SD, UK; rachel.wong@research.sunderland.ac.uk

**Keywords:** poly (ethylene oxide), pentaerythritol tetra-acrylate, hydrogel, lidocaine hydrochloride, diclofenac sodium, ibuprofen

## Abstract

Novel poly (ethylene oxide) (PEO) hydrogel films were synthesized via UV cross-linking with pentaerythritol tetra-acrylate (PETRA) as cross-linking agent. The purpose of this work was to develop a novel hydrogel film suitable for passive transdermal drug delivery via skin application. Hydrogels were loaded with model drugs (lidocaine hydrochloride (LID), diclofenac sodium (DIC) and ibuprofen (IBU)) via post-loading and in situ loading methods. The effect of loading method and drug physicochemical properties on the material and drug release properties of medicated film samples were characterized using scanning electron microscopy (SEM), swelling studies, differential scanning calorimetry (DSC), fourier transform infrared spectroscopy (FT-IR), tensile testing, rheometry, and drug release studies. In situ loaded films showed better drug entrapment within the hydrogel network and also better polymer crystallinity. High drug release was observed from all studied formulations. In situ loaded LID had a plasticizing effect on PEO hydrogel, and films showed excellent mechanical properties and prolonged drug release. The drug release mechanism for the majority of medicated PEO hydrogel formulations was determined as both drug diffusion and polymer chain relaxation, which is highly desirable for controlled release formulations.

## 1. Introduction

The feasibility of hydrogel film patches as vehicles for transdermal drug delivery was first acknowledged in the late 80’s [1]. Since then, a variety of natural and synthetic polymers such as gelatin, pectin, chitosan, and poly (vinyl alcohol) have been studied for potential application in this area [2,3]. Synthetic polymer poly (ethylene oxide) (PEO) is FDA-approved due to negligible immunogenicity issues and absence of residues or vaporous elements when applied onto skin. Besides, high molecular weight grades of PEO have high viscosity promoting the formation of strong, solid gels for transdermal application.

The fabrication of PEO hydrogels via ultraviolet (UV) radiation is advantageous in terms of health and safety aspects, cost and efficiency; also, UV cross-linked pure PEO hydrogel films have shown significantly stronger mechanical profiles than those obtained from electron beam, with Young’s moduli similar to those of human skin [4]. Due to lack of photosensitive chromophoric groups in PEO, the addition of cross-linkable moieties, such as acrylate groups, is essential to initiate the UV crosslinking process [5]. The principal concern of this cross-linking method is the presence of residual acrylate monomer. However, the inclusion of pentaerythritol tetraacrylate (PETRA) as cross-linking agent was evaluated to be safe for dermatological application as long as it is within the maximum acceptable limit of 10% *w*/*w* [6].

The ability of hydrogel film formulations to deliver a drug transdermally via passive diffusion typically relies on its inherent network properties, drug-polymer interactions, amount of entrapped drug, and drug solubility. To the best of our knowledge, a detailed description of these on PEO films has not been reported before. Therefore, the aims of this work are: (1) to explore different drug loading methods onto PEO hydrogel films and their effect on the properties of resultant formulations; (2) to investigate the effect of hydrophilic and lipophilic model drugs: lidocaine hydrochloride (LID), diclofenac sodium (DIC), and ibuprofen (IBU) on the microstructure of PEO hydrogel films; and (3) to characterize the passive permeation profiles of selected drug loaded PEO hydrogel formulations via diffusion studies. LID and DIC were chosen as hydrophilic/ionized model drugs and IBU was chosen as a lipophilic model drug.

LID is one of the most commonly administered local anesthetics due to its efficacy, reliability and low toxicity. However, this hydrophilic drug often exists in ionized form, causing it to be rarely applied percutaneously due to its poor penetration through intact skin. The non-steroidal anti-inflammatory drug (NSAID) DIC is one of the most frequently prescribed drugs, possessing anti-inflammatory, analgesic and anti-pyretic properties. Since it is highly water insoluble, salt forms are often preferred in commercial formulations to avoid solubility problems. Similarly, IBU is one of the best tolerated NSAID, despite its low water solubility and poor gastrointestinal absorption. Due to the aforementioned solubility reasons and systemic side effects, the transdermal administration of NSAIDs has been studied using various formulation approaches [7]. At present, Voltarol^®^ is the only NSAID patch available in the UK. This medicated plaster marketed as gel patch, contains 1% *w*/*w* diclofenac epolamine and is licensed for local symptomatic treatment of pain in epicondylitis and ankle sprain [8]. The physicochemical properties of all model drugs are summarized in Table 1.

## 2. Materials and Methods

### 2.1. Materials

Polyethylene oxide (PEO) (${\overset{̿}{M}}_{w}$ ≈ 1,000,000 g/mol), pentaerythritol tetraacrylate (PETRA), Lidocaine hydrochloride (LID), and Diclofenac Sodium (DIC) were purchased from Sigma-Aldrich, (Dorset, UK). Ibuprofen (IBU) was from Knoll Pharmaceuticals (Nottingham, UK). All materials were used as received. Distilled water (Triplered, Long Crendon, UK) was used for dissolving PEO and model drugs, and swelling of PEO hydrogel films.

### 2.2. Synthesis of PEO Hydrogel Films

8 g of PEO were premixed in distilled water (100 mL) under intensive stirring (IKA ^®^Werke GmbH & Co. KG, Straufen, Germany) for 24 h until a homogeneous gel like mixture was formed. PETRA was then added into the premixed gel, and the entire mixtures were further stirred for 8 h until homogeneous. The cross-linker concentration was fixed at 5% *w*/*w*, as this concentration was found to be optimum in producing gel films with good mechanical properties [4], as well as being safe for dermatological application [6]. The viscous mixture was allowed to stand for 16 h to remove entrapped air bubbles before it was cast onto a glass tile (dimension = 30 cm × 30 cm). The cast gel was dried in a fume cupboard at room temperature (22 °C) for two days to form a xerogel film. The thickness of the xerogel film was measured using a micrometer (Duratool, Taichung, Taiwan), and was found to be 200 μm. The circular film was then cut into smaller rectangular pieces (3 cm × 5 cm).

### 2.3. UV Irradiation of PEO Hydrogel Films

The irradiation process was carried out using a 150 W medium pressure mercury lamp (TQ 150 Heraeus Noblelight GmbH, Kleinostheim, Germany, UV emission = 248–579 nm, λ_max_ = 366 nm), installed inside a quartz jacket within a cylindrical quartz tube. Each piece of film was individually fixed on the inner glass wall of the UV reactor, with approximately 4 cm directly below the head for perpendicular irradiation to achieve optimal light intensity. The total irradiation time was 11 min under a flow of nitrogen. The films were turned upside down after 5.5 min.

### 2.4. Drug Loading

Model drugs, LID, DIC, and IBU were loaded into the hydrogel films via two different methods [16].

#### 2.4.1. Post-Loading

Six replicates of unmedicated dry hydrogel films were immersed in 25%, 50%, 75%, and 100% saturated model drug solutions at 32 °C for 24 h. LID and DIC solutions were prepared using distilled water as solvent, while the solvent for IBU solution was ethanol water (50:50). Preliminary studies showed that 32 °C and 24 h were the optimum conditions to ensure maximum drug load. The fully saturated drug solution concentrations at 32 °C were 9.13% *w*/*v* for LID, 1.98% *w*/*v* for DIC, and 8.01% *w*/*v* (in 50% *w*/*v* ethanol solution) for ibuprofen. After 24 h, the loaded hydrogels were weighed and placed in the vacuum oven for 24 h to dry completely. The dried xerogel films were then weighed and the drug load was determined by using Equation (1). This equation was adapted from Peppas et al., as the weight of drug was taken into account [17].

(1) $$\mathrm{Drug}\;\mathrm{Loading}\;\left(\%\right)=\left(\;\frac{M_{D}-M_{0}}{M_{0}}\;\right)\;\times \;100\%$$

where *M***_0_** is the initial weight of xerogel and *M***_D_** is the weight of drug-loaded xerogel.

#### 2.4.2. In Situ Loading

The calculated amount of model drugs (lidocaine and diclofenac) were weighed and initially dissolved in distilled water. PEO powder was then added to the solution under intensive stirring, as previously described in Section 2.2. In order to compare both loading methods, the exact amount of model drug was included in the mixture, based on the corresponding drug load obtained from immersions in 25%, 50% 75% and 100% saturated drug solutions, as described in post-loading method. Ibuprofen was not loaded via this method, as major phase separation was observed from the synthesized drug loaded hydrogel films due to its hydrophobic nature.

### 2.5. Scanning Electron Microscopy

The morphology of drug loaded hydrogel samples fabricated by both loading methods was evaluated using a scanning electron microscope (Hitachi, Tokyo, Japan) operated in high vacuum mode at an accelerating voltage of 5 kV. The swollen hydrogel samples were transferred into liquid nitrogen for 10 min, and freeze-dried in an ALPHA 2-4/LSC device (Martin Christ Gefriertrocknungsanlagen GmbH, Osterode am Harz, Germany) under a vacuum of 0.1 Pa at −70 °C for 48 h to thoroughly remove the water. The freeze-dried hydrogel samples were put into liquid nitrogen for a sufficient length of time, fractured with a razor blade to expose the internal structures, and stuck onto the sample holder. All samples were sputter-coated with gold for 2 × 10^5^ s before observation.

### 2.6. Differential Scanning Calorimetry

Differential scanning calorimetry (DSC) of pure compounds and hydrogel samples was performed using a DSC Q1000 (TA instrument, New Castle, DE, USA) calibrated with indium standard. Film samples (2–4 mg) were accurately weighed using a microbalance (Mettler-Toledo, Greifensee, Switzerland), placed in an aluminium hermetic sample pan and sealed with a lid. The samples were subjected to standard heat/cool/heat cycles (heating and cooling rate were 10 and 5 °C/min respectively), under a constant flow of nitrogen (50 mL/min). Samples were subjected to first heating past expected melting points in order to examine the material in ‘as received’ condition, then cooled and reheated from a temperature below *T*_g_ to above melting point in order to investigate crystalline to amorphous transitions. The heating and cooling temperatures applied for each sample type is summarized in Table 2. All DSC runs were performed in triplicate. The first heating cycle is represented as cycle 1, cooling as cycle 2, and second heat as cycle 3. The second heating cycles of these runs were used to determine the glass transition temperature (*T*_g_).

The effect of loaded drugs on the crystallinity of the xerogel’s polymeric network was assessed based on the percentage crystallinity ($X_{c}$) calculation, as shown in Equation (2) [18].

(2) $$X_{c}\;\left(\%\right)\;=\;\frac{\Delta H_{f}\left(T_{m}\right)}{\Delta H_{f}\left(T_{m}{}^{o}\right)}\;\times \;100\%$$

where $\Delta H_{f}\left(T_{m}\right)$ is the enthalpy of fusion measured at melting point *T*_m_ from second heat, and $\Delta H_{f}\left(T_{m}{}^{o}\right)$ is the enthalpy of fusion of a 100% crystalline polymer measured at melting point equilibrium melting point $T_{m}{}^{o}$ (196.8 J/g for PEO) [19].

### 2.7. Fourier Transform Infrared Spectroscopy

Fourier Transform Infrared Spectroscopy (FT-IR) was performed to verify the presence of drug in the hydrogel film and to investigate possible interactions with PEO. FT-IR absorption spectra of pure compounds and xerogel film samples were obtained using a Shimadzu IRAffinity-1S Fourier Transform Infrared Spectrometer (Shimadzu UK Ltd., Milton Keynes, UK) at room temperature. The spectral range was 4000–550 cm^−1^ and the resolution was 2 cm^−1^.

### 2.8. Swelling Measurements

Four replicas of pre-weighed in situ loaded xerogel discs (10 mm diameter) from each sample batch were swollen to equilibrium in 500 mL distilled water for 72 h at 25 °C. For accurate measurements, the swelling medium was replaced daily to ensure complete removal of loaded drugs. The equilibrated hydrogel discs were removed from water, blotted with filter paper, and weighed. The equilibrium swelling of each sample was calculated using Equation (3), which was adapted from Omidian et al., as the weight of drug was taken into account [20].

(3) $$\mathrm{Equilibrium}\;\mathrm{Swelling}\;\left(\%\right)=\;\frac{M_{s}-(M_{D}-\;D)}{\left(M_{D}-D\right)}\;\times \;100\%$$

where $M_{s}$ is the weight of swollen gel at equilibrium and *D* is the weight of loaded drug.

At the end of the swelling experiment, the thickness of the swollen gel discs were measured and recorded. The samples were dried in a vacuum oven until constant weight values were attained and reweighed. The gel fraction was calculated using the following equation, which was adapted from Yoshii et al., with weight of drug taken into account [21].

(4) $$\mathrm{Gel}\;\mathrm{Fraction}\;\left(\%\right)=\;\frac{M^{\prime }}{\left(M_{D}-D\right)}\;\times \;100\%$$

where *M’* refers to the weight of xerogel after extraction of water.

#### Determination of Average Molecular Weight between Cross-Links, Cross-Linking Density, and Mesh Size

The calculation of average molecular weight between two adjacent cross-links (${\bar{M}}_{c\;}$), cross-linking density ($\rho_{c}$), and mesh size (ξ) of all PEO hydrogel formulations was based on the equilibrium swelling theory, assuming Gaussian distribution of cross-linked polymer chains. Since the non-ionic hydrogel samples were cross-linked in a dry state, the following equation suggested by Flory and Rehner was used to estimate ${\bar{M}}_{c\;}$ [22]:

(5) $$\frac{1}{{\bar{M}}_{c\;}}=\;\frac{2}{{\bar{M}}_{n}}-\;\frac{\left(\frac{\bar{\upsilon \;}}{V_{1}}\right)[\ln\left(1-V_{2,s}\right)+V_{2,s}+X_{1}\left(V_{2,s}{)}^{2}\right]}{[\left(V_{2,s}{)}^{\frac{1}{3}}-\frac{V_{2,s}}{2}\right]}$$

where ${\bar{M}}_{n}$ is the number average molecular weight of the polymer, $\bar{\upsilon \;}$ is the specific volume of polymer (i.e., the reciprocal of polymer density, $\bar{\nu \;}$ = 0.833 cm^3^/g for PEO), $V_{1}$ is the molar volume of water (18.1 cm^3^/mol), $V_{2,s}$ is the polymer volume fraction, and $X_{1}$ is the polymer-solvent interaction parameter (for water-PEO interaction, $X_{1}$ = 0.45 at ~25 °C [23].

${\bar{M}}_{n}$ was calculated using the molar mass dispersity (*Đ*):

(6) $$Đ=\;\frac{{\overset{̿}{M}}_{w}}{{\bar{M}}_{n}}$$

*Đ*, formerly known as the polydispersity index (PDI), is defined as a measure of dispersion of macromolecular species in a sample polymer [24]. *Đ* of PEO ($\overset{̿}{M}$_w_ ≈ 1,000,000 g/mol) was not provided by Sigma Aldrich, but was determined using size exclusion chromatography by Stokes [25], and was found to be approximately 1.8.

The polymer volume fraction ($V_{2,s}$), was determined as follow [26]:

(7) $$V_{2,s}={\left[1+\;\frac{\rho_{p}}{\rho w}\left(\frac{M_{s}}{\left(M_{D}-D\right)}\;-1\right)\right]}^{-1}$$

where $\frac{M_{s}}{\left(M_{D}-D\right)}$ is the weight swelling ratio of swollen hydrogel at equilibrium, $\rho_{p}$ is the polymer density (1.2 g/cm^3^ for PEO) [26], and ρw is the solvent density (1.00 g/cm^3^ for water).

Subsequently, the cross-linking density ($\rho_{c}$) was calculated using $\bar{M_{c}}$ [20,27]:

(8) $$\rho_{c}={\left(\bar{\nu \;}.\;{\bar{M}}_{c}\right)}^{-1}$$

The theoretical mesh size (ξ) of hydrogel samples was estimated according to Equation (9) [27].

(9) $$\xi ={\left(V_{2,s\;}\right)}^{-\frac{1}{3}}{\left(\;\frac{2\;C_{n}{\bar{M}}_{c}}{M_{r}}\;\right)}^{\frac{1}{2}}\;l$$

where *C*_n_ is the polymer characteristic ratio (4.1 for PEO) [20], *M*_r_ is the molecular weight of the repeating units of the composed polymer (44 g/mol for PEO), and l is the average bond length along the polymer chain (1.54 $\dot{A}$ for PEO) [28].

### 2.9. Tensile Testing

Tensile strength and Young’s modulus of film samples were obtained with a Lloyd LS1 Material Tester (AMETEK Test and Calibration Instruments, Largo, FL, USA) at room temperature (22 °C), using a 5.6 N load cell, 21 mm/min preload stress speed, and extension rate of 10 mm/min. Three replicas of unmedicated and drug loaded hydrogel films were cut into rectangular shapes, with a gauge length of 50 mm and width of 10 mm. The samples were clamped and subjected to tension until breakage.

### 2.10. Measurement of Rheological Properties

The rheological properties of unmedicated and drug loaded PEO hydrogel films were determined with a Malvern Kinexus rotational rheometer (Malvern Instruments Ltd., Malvern, UK), equipped with a 20 mm diameter stainless steel parallel plate. Oscillatory measurements were carried to measure the moduli of the films as a function of shear strain (Amplitude Sweep test) and as a function of frequency (Frequency Sweep test). The film sample was fixed between the upper parallel plate and stationary surface, with the gap size set according to individual swollen film thickness (320–470 μm). All tests were performed in triplicate at 32 ± 0.1 °C.

*Amplitude Sweep.* The linear viscoelastic region (LVR) of all samples was determined with an amplitude sweep at incremental shear strains (1 to 10^6^ Pa) and a fixed frequency of 1 Hz. Frequency sweep test was performed subsequently after identifying the appropriate stress and strain values, which were within the field of LVR.

*Frequency Sweep*. Frequency sweep measurements of film samples were carried out at decreasing oscillating frequencies from 100 to 0.1 rad/s Hz. The mean elastic modulus (*G*’) and viscous modulus (*G*’’) were plotted vs. frequency.

### 2.11. *In Vitro* Drug Permeation Studies

#### 2.11.1. pH Analysis

Three replicates of unmedicated and selected formulations containing the highest drug load from both loading methods were initially swollen with distilled water, and their apparent pH values were measured using a Jenway 3010 pH meter (Cole-Palmer Ltd., Staffordshire, UK). The percentage ionization values of all model drugs present in the formulations were calculated using Equation (10) [29].

(10) $$\mathrm{Ionization}\;\left(\%\right)=\frac{100}{1+\mathrm{anti}\;\log\;\left(\mathrm{pH}-pKa\right)}$$

#### 2.11.2. Release Medium

Drug permeation studies were performed in pH 7.2 phosphate buffer, which was prepared by dissolving phosphate buffer saline tablets in a specified amount of distilled water.

#### 2.11.3. Drug Permeation Studies

Drug permeation studies were performed on selected formulations containing the highest drug load from both loading methods. Four replicates of drug loaded xerogels and one unmedicated xerogel of known weight and dimension (1 cm × 1 cm) were initially swollen and individually sealed between an ethylene-vinyl acetate (EVA) membrane (Co Tran 9702 by 3M Healthcare Ltd., Loughborough, UK) and an impermeable backing layer to form a reservoir device. This was then transferred into a beaker containing 10 mL of pH 7.2 phosphate buffer (20 mL for DIC and IBU loaded hydrogels), with the EVA membrane facing towards the release medium. To avoid evaporation of medium, the beakers were sealed with aluminum foil. The entire study was performed at 32 °C under gentle stirring using a magnetic stirrer. At designated time points, the release medium was completely removed and replaced with fresh phosphate buffer, which had been preheated to 32 °C. Samples were collected at designated time intervals and their corresponding drug concentrations were measured in triplicate using UV-visible spectroscopy. At the end of the study, the hydrogels were removed from the reservoir system and placed in a vacuum oven at 40 °C for 24 h for complete moisture removal. The resultant xerogels were then accurately weighed, and the obtained weights were used to cross check the total percentage drug release using Equation (11), which was adapted from Equation (1).

(11) $$Total\;percentage\;drug\;release\;\left(\%\right)=\left(\frac{M_{D}-\;M_{R}}{Weight\;of\;Loaded\;Drug\;}\;\right)\times \;100\%$$

where *M*_R_ is the weight of xerogel after the release study.

The drug release profiles for all tested formulations were evaluated using the following equations corresponding to different kinetic models:

1. Zero-order kinetics [30]
  (12) $$F=K_{0\;}t$$
  where *F* is the fraction of drug release at time *t* and *K*_0_ is the zero order release constant.
2. First-order kinetics [31]
  (13) $$ln\left(1-F\right)=-K_{1}t$$
  where *K*_1_ is the first order release constant.
3. Higuchi model [32]
  (14) $$F=-K_{2}t^{1/2}$$
  where *K*_2_ is the Higuchi constant.
4. Korsmeyer-Peppas Model [33]
  (15) $$\frac{M_{t}}{M_{\infty }}=K_{3}t^{n}$$
  where $\frac{M_{t}}{M_{\infty }}$ is the fraction of drug release at time *t*, *K*_3_ is the release rate constant, and *n* is the release exponent. The different release mechanisms were characterized using the calculated *n* value. When *n* < 0.5, the diffusion mechanism is quasi-Fickian, *n* = 0.5 is Fickian diffusion, 0.45 < *n* < 1 refers to non-Fickian diffusion, *n* = 1 is case-II transport, and *n* > 1 refers to super case-II transport [34].

### 2.12. Statistical Analysis

Statistical analysis was performed using SPSS 17.0 (SPSS Inc., Chicago, IL, USA) to determine statistical differences. The data obtained from swelling studies, DSC, tensile tests, rheological studies, and permeation studies were analysed by one-way analysis of variance (ANOVA). The subgroup means were compared by post hoc Scheffe’s test. Statistical significance for all tests was set at a probability of *p* < 0.05.

## 3. Results and Discussion

### 3.1. Effect of Drug Physicochemical Properties on the Percentage Loading of Post-Loaded Hydrogels

**Figure 1:**
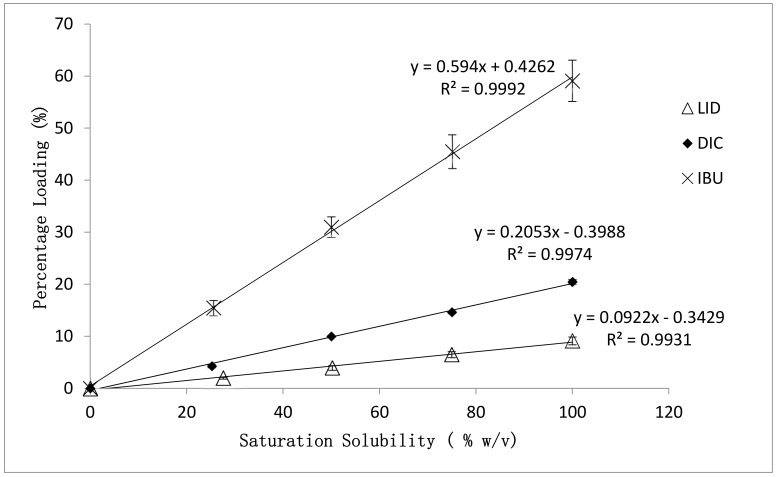
Effect of drug physicochemical properties on the percentage loading of post-loaded hydrogels. Error bars represented the standard deviation (*n* = 6) of samples.

The percentage loading of model drugs into hydrogels obtained from post-loading method was evaluated and summarized in Figure 1. Highly correlated (*R*^2^ > 0.99) linear loading trends were obtained for all model drugs. The highest saturated loading percentage was achieved by IBU (59.08 ± 3.97%), indicating good capability of PEO hydrogels in accommodating hydrophobic compounds. This was followed by DIC (20.68 ± 0.47%) and LID (9.75 ± 0.75%). The calculated standard deviation of loading percentages obtained from all LID and DIC loaded hydrogels were at least 5 times lower than those of IBU loaded hydrogels, indicating a more homogeneous drug load. This is expected, due to the hydrophilic nature of LID and DIC possessing better compatibility with the hydrophilic polymer matrix.

### 3.2. Effect of Loading Method and Drug Physicochemical Properties on the Morphology Characteristics

**Figure 2:**
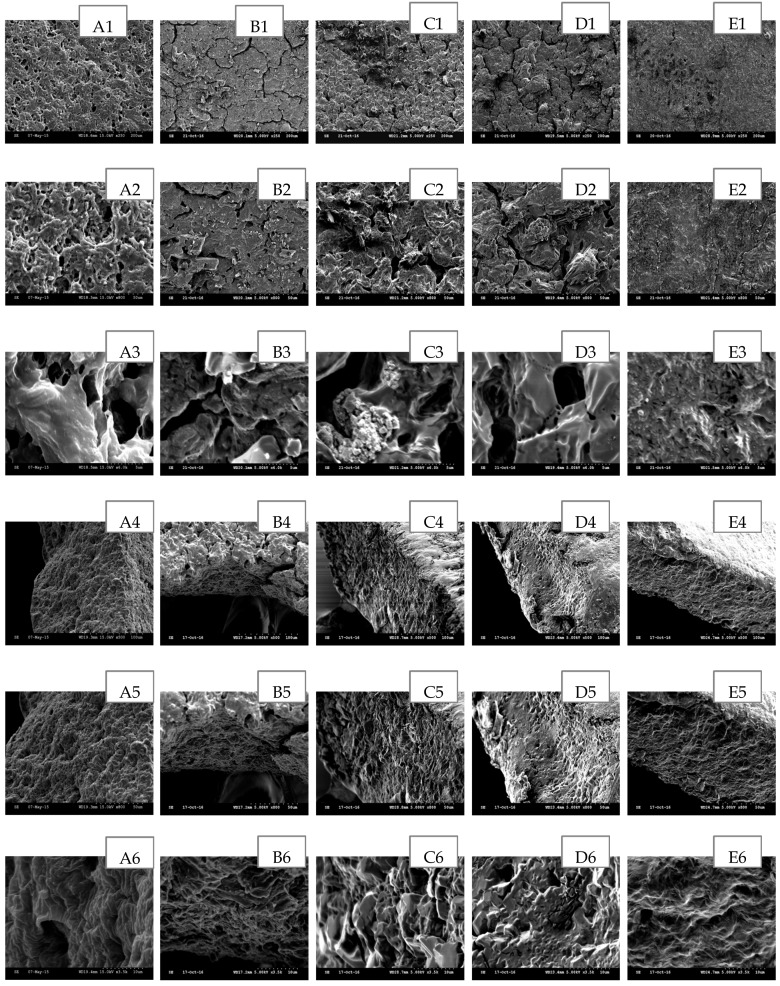
SEM images of post-loaded LID hydrogel network in comparison to unmedicated hydrogel cross-linked with 5% *w*/*w* PETRA. (**A**) Unmedicated PEO, (**B**) PEO-LID 1.18% *w*/*w* (25% saturation), (**C**) PEO-LID 3.64% *w*/*w* (50% saturation), (**D**) PEO-LID 6.31% *w*/*w* (75% saturation), (**E**) PEO-LID 9.75% *w*/*w* (100% saturation). Scale bars are 100, 50 and 5 μm for micrographs featuring the outer surface of hydrogel network (1–3), 100, 50, and 10 μm for micrographs featuring the cross section of hydrogel network (4–6).

**Figure 3:**
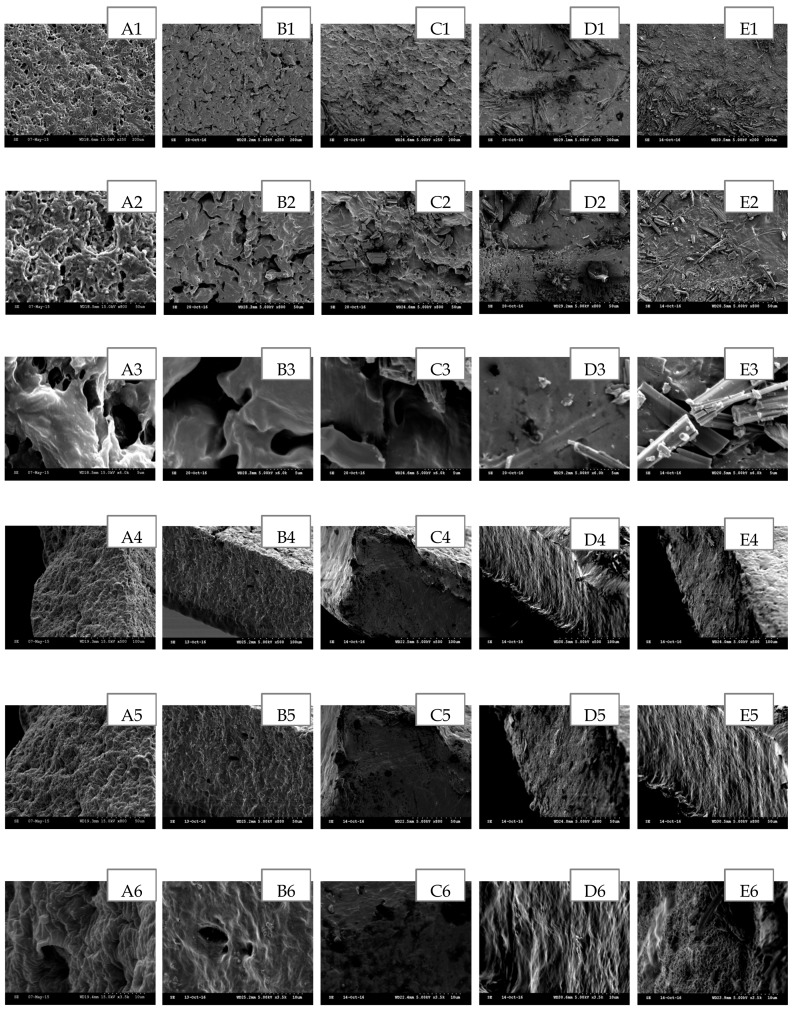
SEM images of post-loaded DIC hydrogel network in comparison to unmedicated hydrogel cross-linked with 5% *w*/*w* PETRA. (**A**) Unmedicated PEO, (**B**) PEO-DIC 3.28% *w*/*w* (25% saturation), (**C**) PEO–DIC 8.39% *w*/*w* (50% saturation), (**D**) PEO-DIC 13.62% *w*/*w* (75% saturation), (**E**) PEO-DIC 20.68% *w*/*w* (100% saturation). Scale bars are 100, 50 and 5 μm for micrographs featuring the outer surface of hydrogel network (1–3), 100, 50, and 10 μm for micrographs featuring the cross section of hydrogel network (4–6).

**Figure 4:**
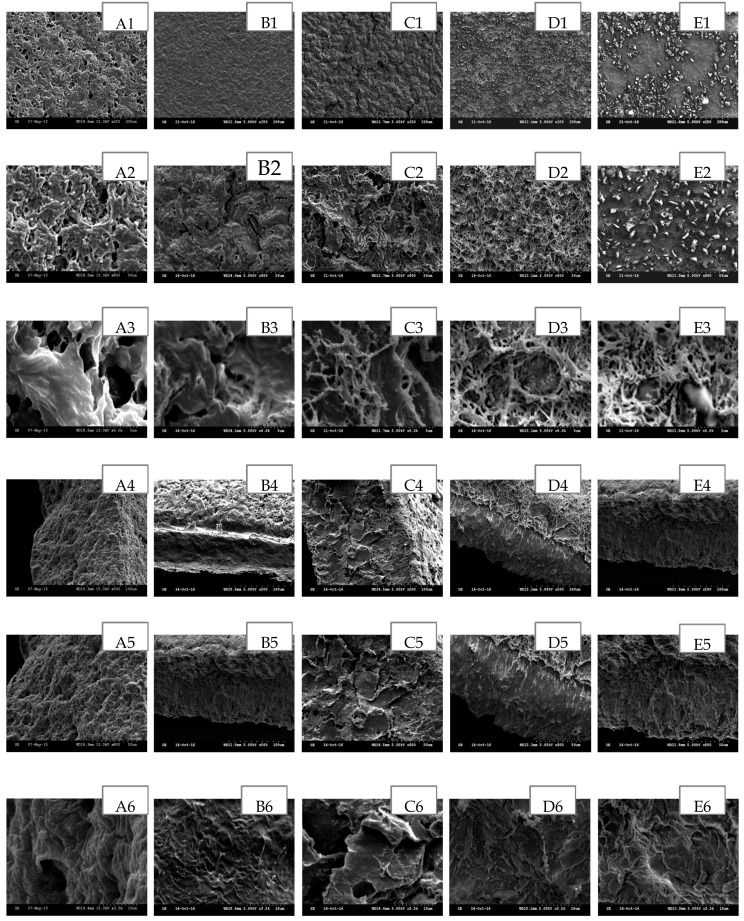
SEM images of post-loaded IBU hydrogel network in comparison to unmedicated hydrogel cross-linked with 5% *w*/*w* PETRA. (**A**) Unmedicated PEO, (**B**) PEO-IBU 15.44% *w*/*w* (25% saturation), (**C**) PEO-IBU 31.01% *w*/*w* (50% saturation), (**D**) PEO-IBU 45.48% *w*/*w* (75% saturation), (**E**) PEO-IBU 59.09% *w*/*w* (100% saturation). Scale bars are 200, 50 and 5 μm for micrographs featuring the outer surface of hydrogel network (1–3), 100, 50, and 10 μm for micrographs featuring the cross section of hydrogel network (4–6).

**Figure 5:**
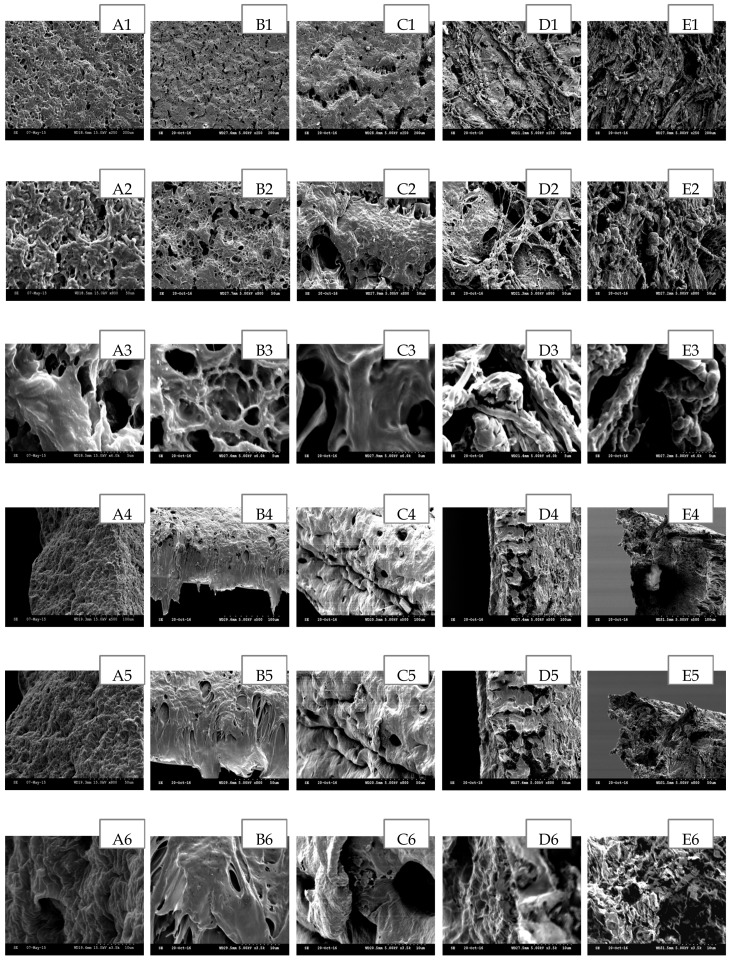
SEM images of in situ loaded LID hydrogel network in comparison to unmedicated hydrogel cross-linked with 5% *w*/*w* PETRA. (**A**) Unmedicated PEO, (**B**) PEO-LID 1.12% *w*/*w*, (**C**) PEO-LID 3.57% *w*/*w*, (**D**) PEO-LID 6.30% *w*/*w*, (**E**) PEO-LID 9.93% *w*/*w*. Scale bars are 200, 50, and 5 μm for micrographs featuring the outer surface of hydrogel network (1–3), 100, 50, and 10 μm for micrographs featuring the cross section of hydrogel network (4–6).

**Figure 6:**
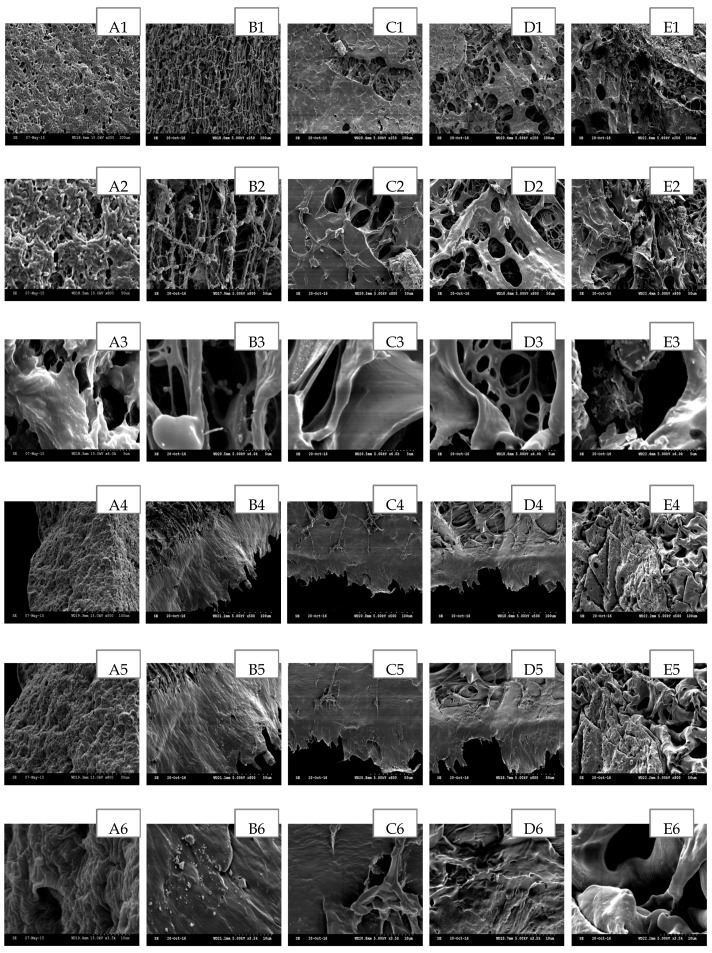
SEM images of in situ loaded DIC hydrogel network in comparison with unmedicated hydrogel cross-linked with 5% *w*/*w* PETRA. (**A**) Unmedicated PEO, (**B**) PEO-DIC 3.17% *w*/*w*, (**C**) PEO-DIC 8.37% *w*/*w*, (**D**) PEO-DIC 13.12% *w*/*w*, (**E**) PEO-DIC 20.26% *w*/*w*. Scale bars are 200 μm, 50 μm and 5 μm for micrographs featuring the outer surface of hydrogel network (1–3), 100 μm, 50 μm, and 10 μm for micrographs featuring the cross section of hydrogel network (4–6).

The scanning electron microscopy (SEM) has been widely used to study the hydrogel’s surface topography and its characteristic network structure [35]. Figure 2, Figure 3, Figure 4, Figure 5 and Figure 6 show the scanning electron micrographs of medicated PEO hydrogel network loaded via both methods in comparison to the unmedicated hydrogel network. From Figure 2, Figure 3 and Figure 4 (images B–E), it can be visually observed that all post-loaded formulations were homogeneously loaded. The model drugs were loaded by first occupying the existing interconnected microporous spaces of the hydrogel network. As the drug saturation increased, more drugs were found deposited onto the film surface. The small amorphous LID particles (Figure 2, images B2–E2) were found sticking together to form bigger particles. This is especially obvious at the highest load (Figure 2, images E1–E3). Both DIC (Figure 3, images B2 and C2) and IBU (Figure 4, images B2–D2) initially appeared as amorphous forms when loaded in lower saturation (up to 50% saturation for DIC and 75% saturation for IBU). At saturated loading, rod-shaped crystals and smooth spherical aggregates were observed for DIC (Figure 3, images D3 and E3) and IBU (Figure 4, images E1–E3) respectively. The crystalline drug was non-homogeneously distributed, and is identified as diclofenac acid from DSC and FT-IR. Overall, the sponge like structure of unmedicated hydrogels was retained when films were post-loaded (Figure 2, Figure 3 and Figure 4, images B4–E4). In contrast, the morphologies of films were distinctively different when drugs were loaded in situ (Figure 5 and Figure 6, images B–E). The model drugs were also found embedded on the polymeric network instead of microporous spaces. Presence of LID (Figure 5, images B3–E3) generated more micropores, and a gradual increment on the mesh size was observed as the drug load increased. Moreover, the amorphous structure of LID was also found to appear differently from the post-loaded ones. For the case of in situ loaded DIC hydrogels (Figure 6, images B–E), films containing 3.17% *w*/*w* DIC (Figure 6, images B1–B3) possessed significantly larger mesh size when compared to the unmedicated ones (Figure 6, images A1–A3). Hydrogels loaded with 8.37% *w*/*w* (Figure 6, images C1–C3), 13.12% *w*/*w* (Figure 6, images D1–D3), and 20.25% *w*/*w* (Figure 6, images E1–E3) DIC were found to be non-homogeneously cross-linked, as irregular distributions of micropores were showcased. Furthermore, their corresponding cross section micrographs featured absence of sponge-like structure (Figure 6, images C4–E6), indicating scarcity of cross-links within the gel network. No rod-shape crystals were observed at higher drug load (Figure 6, images D1–E3) indicating that the drug was present in an amorphous state.

### 3.3. Effect of Drug Physicochemical Properties on the Swelling Properties of In Situ Loaded Hydrogels

The polymeric networks of all in situ loaded formulations were compared to the unmedicated ones via swelling studies. The resultant findings were tabulated in Table 3. It should be noted that swelling studies were not performed on the post-loaded formulations, as similar morphologies to the initial unmedicated film was observed from SEM. From Table 3, it can be seen that in situ drug loading of hydrogels causes marked changes to the gel network, as the obtained equilibrium swelling percentages, ${\bar{M}}_{c}$, $\rho_{c}$, and ξ of all medicated films (except those containing 1.12% *w*/*w* LID) were significantly different from the unmedicated film. This also means that 1.12% *w*/*w* LID was insufficient to cause significant changes on the network properties of blank PEO gels. Nonetheless, all in situ loaded formulations (except those loaded with 13.12% and 20.26% *w*/*w* DIC) fall under the same gel class as the unmedicated gel, which were non-porous, with mesh size (ξ) values between 1 and 10 nm [4]. In situ loaded gels containing 13.12% and 20.26% *w*/*w* DIC exhibited similar network properties as PEO gels cross-linked with 1% *w*/*w* PETRA [4]. The increase of LID and DIC concentration reduced the cross-linking of the gel network, indicating possible interaction between drug and excipients. The large standard deviations obtained from the swelling parameters of in situ loaded DIC hydrogels correlated well with SEM observations of being non-homogeneously cross-linked.

### 3.4. DSC Analysis

**Figure 7:**
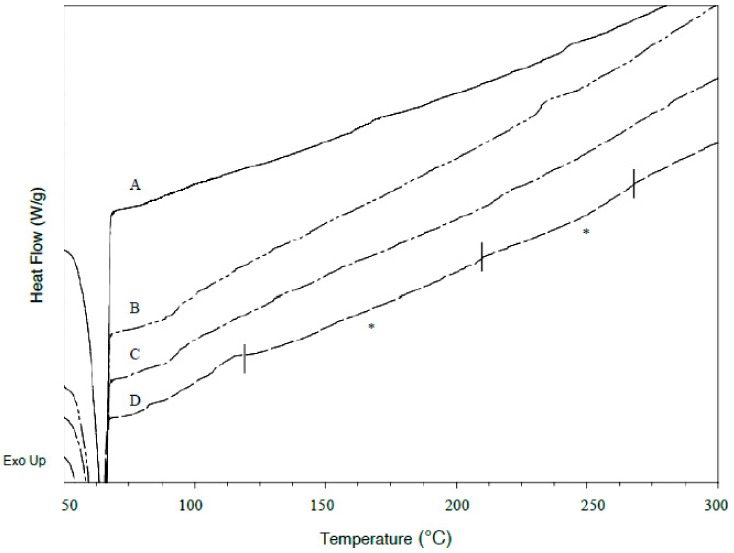
DSC thermograms of post-loaded DIC formulations. (**A**) PEO-DIC 3.28% *w*/*w* (25% saturation), (**B**) PEO-DIC 8.39% *w*/*w* (50% saturation), (**C**) PEO-DIC 13.62% *w*/*w* (75% saturation), (**D**) PEO-DIC 20.68% *w*/*w* (100% saturation). Melting endotherms are marked with asterisks (*).

**Figure 8:**
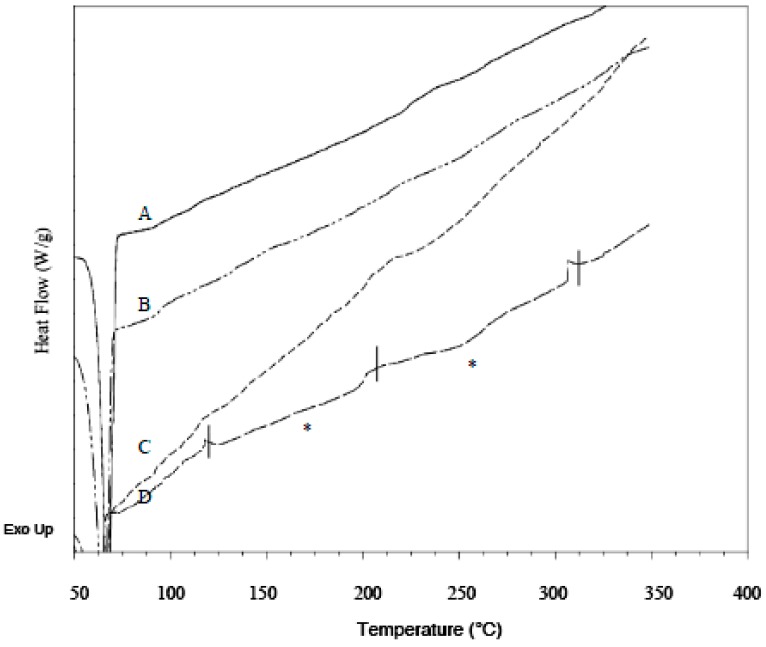
DSC thermograms of in situ loaded DIC formulations. (**A**) PEO-DIC 3.17% *w*/*w*, (**B**) PEO-DIC 8.37% *w*/*w*, (**C**) PEO-DIC 13.12% *w*/*w*, (**D**) PEO-DIC 20.26% *w*/*w*. Melting endotherms are marked with asterisks (*).

DSC was performed on pure drugs and PEO to establish their thermal parameters, and on unmedicated and drug loaded xerogel films to determine the presence of crystals in the films. For pure PEO, the average melting temperature (*T*_m_) obtained from first and second heat was 69.1 ± 0.2 and 65.2 ± 0.2 °C respectively, with melting enthalpy of fusion $\left(\Delta H_{f}\right)$ respectively being 191.7 ± 3.3 and 123.7 ± 4.7 J/g. The lower polymer melting endotherm observed in second heat indicated a small decrease in polymer crystallinity. In addition, the polymer *T*_g_, which was expected to occur between −50 to −57 °C [36] was not found present in corresponding thermograms. Similar observation to this had been recorded in literature [37,38,39]. Reason for it might be the high crystallinity of samples, as high molecular weight PEO are highly crystalline thermoplastics [40]. After cross-linking, the *T*_m_ from first and second heating of resultant unmedicated films respectively occurred at 65.3 ± 0.2 and 63.1 ± 0.9 °C when heated up to 80 °C. The films were also heated up to 350 °C, and their *T*_m_ from first and second heating respectively occurred at 69.7 ± 0.8 and 57.0 ± 0.5 °C. The *T*_m_ difference is found to be statistically significant, indicating thermal behaviours of PEO hydrogels were significantly affected by different thermal conditions. Hence, both values of *T*_m_ were assigned as standard references. Pure LID exhibited a sharp endothermic peak with *T*_m_ at 78.4 ± 0.2 °C when first heated. The drug remained amorphous after the first melt, as no melting point was detected at second heat, and a subtle *T*_g_ occurred at 32.9 ± 0.2 °C, which is in agreement with literature *T*_g_ value [41]. The first heating of pure DIC showed *T*_m_ at 288.2 ± 0.7 °C, followed by an exothermic event at 306.4 ± 0.8 °C and decomposed immediately at 320.92 ± 0.7 °C. Hence, no *T*_m_ and *T*_g_ were detected for DIC during second heat. The highly crystalline IBU exhibited sharp *T*_m_ at 75.5 ± 0.1 °C when first heated, and then turned semi crystalline, with *T*_g_ and *T*_m_ detected during second heat, at −42.7 ± 0.3 and 75.6 ± 0.2 °C respectively. The complete disappearance of characteristic pure drug melting endotherms in all LID and IBU formulations loaded via both loading methods verified that the different drug morphologies observed from SEM were amorphous. As for DIC formulations, the presence of subtle endothermic peaks at approximately 165 and 255 °C (melting endotherms are marked with asterisks in Figure 7 and Figure 8) indicated two forms of diclofenac, respectively referring to its acidic and salt form. Due to the irregularity in shape, the actual melting points of the polymorphs could not be precisely determined. The conversion of drug to its acidic form was confirmed based on the melting endotherm being close to the literature melting point diclofenac acid (156–158 °C) [42], and presence of carboxylic acid functional groups identified from FT-IR. The rod-shaped crystals present in the SEM micrograph of formulations post-loaded with 20.68% *w*/*w* DIC was also identified as diclofenac acid by FT-IR. Comparing between loading methods, the amount of drug transformed into its acidic and salt crystals was deemed higher for in situ loaded formulations, as the corresponding melting endotherms were more obvious.

The typical thermograms obtained from first (cycle 1) and second (cycle 3) heating for formulations containing the highest load for each model drug were respectively shown in Supplementary Figures S1 and S2, with comparsion to those obtained from unmedicated ones and corresponding pure drug. In order to rule out the possibility of ethanol (EtOH) instead of drug affecting the polymeric network, blank hydrogel samples were subjected to the same process involved in the post loading of IBU but without presence of drug, and the thermal profile of the resultant films was used as control for IBU loaded formulations.

The polymer’s melting endotherm obtained from first and second heat was very similar in all formulations except for DIC formulations, where the melting endotherm from second heating was sharper compared to the first heat. Hence, the crystallinity of the polymer network was accounted based on second heat. The effect of loaded drugs on the crystallinity of polymeric network of xerogel was assessed based on the percentage crystallinity ($X_{c}$) calculation, as shown in Equation (2) and the findings are summarized in Table 4. The *T*_m_ and %*X*_C_ of our unmedicated xerogel cross-linked with pentaerythritol tetra-acrylate (PETRA) were found to be similar to PEO hydrogels synthesised by Doytcheva et al. that were cross-linked with 5% *w*/*w* pentaerythritol triacrylate (PETA) [5]. EtOH treatment resulted in significant decrease in blank films %X_C_, but the change in crystallinity portion was not prominent enough to cause significant depression on its corresponding *T*_m_. Similar trend was also observed in all post-loaded LID formulations. The drug-polymer interaction was more obvious in post-loaded DIC and IBU xerogel formulations, as significant decrease of %X_C_ led to significant depression of *T*_m_, even at lower loads. All in situ loaded LID formulation possess similar %X_C_ and *T*_m_ as corresponding unmedicated ones, while the reductions in %X_C_ and *T*_m_ were significant in formulations in situ loaded with 13.12% *w*/*w* DIC and above. This concluded that the crystallinity of PEO hydrogel network was more likely to be retained when drugs were loaded in situ, as was also confirmed by FTIR (see Section 3.5).

### 3.5. FT-IR Analysis

#### 3.5.1. Chemical Identification

**Figure 9:**
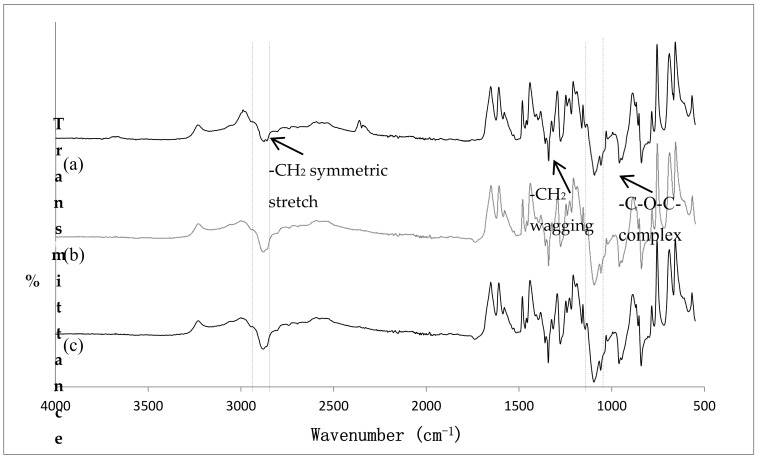
FT-IR spectra of (**a**) pure PEO, (**b**) unmedicated PEO xerogel film, (**c**) EtOH treated unmedicated PEO xerogel film.

The FT-IR of pure PEO, unmedicated PEO xerogel film, and EtOH treated unmedicated xerogel films are illustrated in Figure 9. The characteristic peaks of PEO were assigned based on generally accepted spectral interpretation [43,44]. Most of the characteristic peaks of pure PEO were attributed to methylene (CH_2_) group, with related peaks occurring at 2877.79 cm^−1^ (–CH_2_ symmetric stretching), 1467.83 cm^−1^ (–CH_2_ scissoring), 1359.82 and 1342.46 cm^−1^ (–CH_2_ wagging), and 1276.88 cm^−1^ (CH_2_ twisting). This confirmed that PEO possessed a certain extent of hydrophobic properties, allowing the loading of IBU through hydrophobic interactions. Although PEO is typically considered as hydrophilic, its hydrophobic properties have been documented [45,46]. The appearance of the two previously mentioned –CH_2_ wagging peaks confirmed the high crystallinity of PEO [47]. Besides, the spectra revealed presence of –C–O–C– absorption complex, as an apparent triplet peak occurring at 1143.78, 1095.57 (maximum intensity), and 1058.92 cm^−1^ was identified. This complex, resulting from the combination of ether group and methylene group, is very sensitive to polymer chain conformational changes [48]. Hence, special attention was paid to its shape, intensity and shift for the detection of possible interaction. It should be noted that the peak present at 2349.30 cm^−1^ was due to atmospheric carbon dioxide [49]. The spectra of cross-linked unmedicated PEO xerogel film showed similar characteristic peaks as pure PEO with no additional peaks present. The intensity of –CH_2_ symmetric stretching peak was slightly increased after cross-linking. Although the crosslinking of PEO via gamma radiation was thought to widen and reduce the intensity of –C–O–C– complex [48], our results have shown to be otherwise. This may be due to incorporation of PETRA in the UV cross-linking process. In addition, two peaks of the triplet peak shifted from 1142 to 1144 cm^−1^, and 1094 to 1096 cm^−1^. This suggested an increase in inter- and intra-molecular PEO interactions, most likely due to polymer chains entanglement when PEO was premixed in distilled water during the synthesis of hydrogel films [48]. For the case of EtOH-treated unmedicated film, the –CH_2_ symmetric stretching slightly shifted to higher wavenumbers (2881.65 cm^−1^); this indicated weakening of hydrogen bond between polymer chains, which may contribute to the small decrease in film crystallinity as observed in DSC.

Pure LID showed characteristic peaks at 3450.65 and 3383.14 cm^−1^ (amide N–H stretching), 1654.92 cm^−1^ (amide C=O stretching), and 1541.12 cm^−1^ (amide N–H bending) [50]. Besides, the peaks located in the regions between 2300 and 2600 cm^−1^ were assigned to N–H vibrations of hydrochloride salt [51]. Pure DIC exhibited distinctive peaks at 3388.93 cm^−1^ (secondary amine N–H stretching), 1571.99 cm^−1^ (carboxylate COO^−^ asymmetric stretching), 1556.55 cm^−1^ (dichlorophenyl ring), 1498.69 cm^−1^ (phenyl acetate), and 1396.463 cm^−1^ (carboxylate COO^−^ symmetric stretching) [52]. FT-IR absorption spectra of pure IBU revealed characteristic peaks at 2922.16 cm^−1^ (carboxylic acid O–H stretching) and 1705.07 cm^−1^ (carboxylic acid C=O stretching) [53].

#### 3.5.2. Effect of Loading Method and Drug Physicochemical Properties on the Drug-Polymer Compatibility

The FT-IR absorption spectra of all medicated xerogel films loaded via both drug-loading methods in comparison to corresponding blank film and model drug are shown in Supplementary Figures S3–S7. Presence of drug was confirmed in all formulations as their respective spectra revealed characteristic peaks of loaded model drug. These peaks appeared either in separate form, or fused with the characteristic peaks of unmedicated xerogel film. The spectra of post-loaded LID formulations (Supplementary Figure S3) loaded in drug solution of 75% saturation and above, and all of post-loaded IBU formulations (Supplementary Figure S5) were almost identical to the spectra of corresponding model drug. This indicated presence of intact drug on film surface rather than being entrapped into the polymeric network, as also observed using SEM. If the drug is completely entrapped within the polymeric network, its characteristic peaks will not be detected [54]. The drug-polymer interaction for post-loaded LID formulations is clearly detected in the spectra of those with a drug concentration of 3.64 % *w*/*w* (Supplementary Figure S3d). The polymer’s –CH_2_ symmetric stretching merged with the drug’s hydrochloric salt N-H vibrations, resulting in a triplet peak complex with maximum intensity at 2922.16 cm^−1^. In addition, the polymer –C–O–C– complex peak shifted to lower wavenumbers (from 1095.57 to 972.12 cm^−1^ (maximum intensity) respectively) with decreased intensity, indicating presence of inter-molecular hydrogen bonding. Furthermore, the reduced –CH_2_ wagging peak explained the decrease in polymer crystallinity of post-loaded LID formulations. Although the spectra of all post-loaded DIC formulations (Supplementary Figure S4) appeared to be a combination of pure drug and unmedicated xerogel film, the presence of additional peaks at 3645.47 and 3342.64 cm^−1^ (O–H stretching), and 1734.01 cm^−1^ (carboxylic acid C=O) confirms the conversion of active drug from salt form to acid form. In addition, FT-IR study was specifically performed on the rod-shaped crystals present in formulations post-loaded with 20.68% *w*/*w* DIC, as observed from SEM. These crystals were identified as diclofenac acid, due to the appearance of O–H and C=O stretching vibrations in their corresponding FT-IR spectra. The observation also verifies the different diclofenac polymorphs observed from SEM. Due to the size of these peaks, it is concluded that the amount of drug in acid form present in these formulations is fairly low. This correlates well with the subtle thermal events corresponding to diclofenac acid as observed from DSC. As the drug load increases, diclofenac acid’s characteristic carboxylic acid C=O gradually shifted to higher wavenumber (corresponding peak shifted to 1737.86 cm^−1^) with decreasing intensity, which indicated weakening of inter-molecular hydrogen bonding. In addition, the gradual reduction and shifting towards lower wavelength from drug’s characteristic secondary amine N–H stretching peak and the polymer –C–O–C– complex (from 3388.93 to 3383.14 cm^−1^ and from 1095.57 to 1093.64 cm^−1^ (maximum intensity) respectively) with increasing drug load also suggested presence of inter-molecular hydrogen bonding. Similar observation was found in IR spectra of post-loaded IBU formulations, as additional peaks occurred at 3450.65 and 3385.07 cm^−1^. The increased intensity and shifting of drug characteristic carboxylic acid C=O stretching towards higher wavenumbers (from 1705.07 to 1712.79 cm^−1^) and the decreased intensity and shifting of polymer –C–O–C– complex peak towards lower wavenumbers (peak with maximum intensity shifted from 1095.57 to 1072.42 cm^−1^) for formulations with lower loading provide strong evidence of hydrogen bonded IBU with PEO film. Comparison between post-loaded and in situ loaded formulations, showed that the in situ loading method provides better drug entrapment within the hydrogel network due to absence of spectra same or near identical to pure drug. The lack of intact drug on film surface of post-loaded formulations is supported by SEM findings. Moreover, the drug-polymer interaction is influenced by the loading method, as different spectral changes were observed between formulations containing the same drug but loaded differently, thus explaining the different polymer crystallinity as observed in DSC. The better resemblance of in situ loaded formulations spectra to the polymer PEO spectrum also implied less drug-polymer interaction, hence verifies the conclusion from DSC analysis regarding the likelihood of polymer crystallinity being maintained when drugs were loaded via in situ loading method. For in situ loaded LID formulations (Supplementary Figure S6), the triplet peak complex with maximum intensity at 2922.16 cm^−1^, observed in the spectra of post-loaded ones, was not present. Instead, the presence of hydrogen bonding was indicated, as the polymer’s –C–O–C– complex shifted to lower wavenumber (peak with maximum intensity shifted from 1095.67 to 1035.74 cm^−1^) with decreasing intensity as the drug load increases. The presence of diclofenac acid was also confirmed in in situ loaded DIC formulations (Supplementary Figure S7), as additional peaks were observed at 3450.62 (O–H stretching) and 1718.58 cm^−1^ (carboxylic acid C=O). These characteristic peaks appeared differently when compared to post-loaded DIC formulations, thus supported the observation of drug morphology being affected by loading method. In addition, the characteristic peaks were also more prominent compared to the ones observed from post-loaded formulations, hence implying the amount of drug converted from salt form to acid form was higher. This is in agreement with the more obvious diclofenac acid melting endotherm observed from DSC. As the loading concentration increases, the peak corresponding to diclofenac acid’s carboxylic acid C=O shifted to higher wavenumbers (from 1718.58 to 1737.86 cm^−1^) with reduced intensity, indicating weakening of inter-molecular hydrogen bonding. Furthermore, the polymer’s –CH_2_ symmetric stretching gradually shifted to lower wavenumber (from 2877.79 to 2875.87 cm^−1^) with increasing intensity, which suggested increase in intermolecular stabilization due to longer chains [55]. This observation verifies the formulations’ higher average molecular weight between crosslink calculated from swelling studies. Overall, the reduction in the formulations’ drug characteristic peaks relative to pure drug implied decrease in drug crystallinity and change into amorphous form [56].

### 3.6. Effect of Loading Method and Drug Physicochemical Properties on the Mechanical Properties

The effect of loaded drugs on the mechanical properties is presented in Table 5. It can be seen that ethanol treatment did not cause significant changes to the mechanical properties of hydrogels. As expected, the tensile strength and percentage elongation of all post-loaded formulations were comparable to the corresponding unmedicated hydrogels, due to morphological similarities. The post-loading of LID did not have an effect on film’s tensile strength but gave a slight increase on their percentage elongation. Therefore, the decrease in corresponding Young’s moduli were insignificant (*p* > 0.05). The effect of post-loaded drugs in reducing the mechanical properties of hydrogel films was more obvious for the case of DIC and IBU formulations. This can be attributed to drug causing significant decrease in the polymer crystallinity. On the other hand, the mechanical properties of in situ loaded formulations were significantly different from unmedicated hydrogels. In situ loaded LID seem to have a plasticizing effect, causing a moderate decrease in formulations’ tensile strength and Young’s modulus, while dramatically improved the percentage elongation (up to 50% improvement). The marked decrease in the mechanical properties of in situ loaded DIC formulations was due to the low cross-linking density resulting in significantly weaker films. Nonetheless, these observations agree with findings from SEM, swelling studies, DSC and FT-IR.

### 3.7. Effect of Loading Method and Drug Physicochemical Properties on the Rheological Properties

The effect of loaded drugs on the rheological properties is summarized in Table 6. No values were presented for 8.3% *w*/*w* and above in- situ loaded DIC formulations, as no linear viscoelastic regions (LVR) were obtained during the study, indicating insufficient cross-linking to form a stable microstructure. This observation agreed with their irregular and scarce gel network observed from SEM and their largely deviated swelling parameters. The lack of cross-link formation in these formulations may be due to chlorine atoms of DIC moiety forming free radicals under UV radiation. This is thought to deactivate the PETRA radicals. As a result, less PEO radicals were generated for cross-linking. From Table 6, it can be seen that all loaded formulations, except those containing 1.18% *w*/*w* post-loaded LID showed significant increase in elastic modulus (*G*’). In addition, the G’ increases with increasing drug load, indicating the increase of undissolved drug present in film [57]. All *G*’ values were obviously higher than viscous modulus (*G*”) indicated all tested films behaved more solid-like. The high *G*’ values in comparison to corresponding *G*” observed in situ loaded LID formulations indicated pronounced elastic deformation under shear stress, which agrees with the films’ high toughness observed from tensile testing. The increase of viscous modulus (*G*”) is thought to be related to the significant decrease in polymer crystallinity, as the increase in polymer chain movement resulted in an increase in flow properties. For both loading methods, the increase of drug concentration generally decreased the microstructure stability. This is reflected through the decrease in critical strain ($\gamma_{0}$) values. $\gamma_{0}$ is known as the critical oscillatory strain a hydrogel film can withstand before bond breakage occurs [58]. The moduli of all tested films were independent of frequency, showing no change in degree of viscoelasticity. This is typical of gel behaviour and indicative of a homogeneous cross-linked network. The complex viscosity ($\eta^{*}$) is a frequency-dependent modulus accounting for both the elastic and viscous properties of a material. The higher the $\eta^{*}$ value, the more solid-like the material is and/or the greater is the resistance to flow [59]. Hence, the relationship between $\eta^{*}$ and drug release rate is expected to be inversely proportional. Increase of drug concentration generally increased the formulations’ $\eta^{*}$ values (Table 6). The in situ loaded LID formulations possessed the highest $\eta^{*}$ values among all, which correlated well with their improved tensile strength and percentage elongation. Overall, all formulations’ $\eta^{*}$ values were found to decrease with increasing oscillatory frequency, thus confirming the shear-thinning behaviour of the tested films [60].

### 3.8. Effect of Loading Method and Drug Physicochemical Properties on the Drug Release Behaviour via a Synthetic Membrane

#### 3.8.1. pH and Drug Ionization

The ionization of drugs was evaluated, as it is expected to significantly impact the transdermal delivery of drugs, in terms of release kinetics, permeability, and absorption via passive diffusion. In most cases, the outermost of skin (stratum corneum) permeability coefficient for non-ionized species of a drug is 1–2 orders of magnitude larger than the permeability coefficient of its ionized counterpart [61]. Table 7 summarized the calculated percentage drug ionization expected in formulations containing drug in salt forms, and in release medium (phosphate buffer pH 7.2), based on formulation experimental pH measurements and literature drug p*K*a values. The applicability of all studied formulations for dermatological use is confirmed, as their apparent pH values were nearly similar to the pH of skin (pH 4.5–6.5) [62]. LID loaded from both loading methods was almost completely ionized, with some of its non-ionized counterpart expected to be present in the release medium. The high ionization of the drug may contribute to the lower hydrogel post-loading capacity, as PEO itself is a non-ionic polymer [63]. In the case of anionic polymer gels, the loading of LID should be higher due to the presence of opposite charges. Contrastingly, almost all loaded DIC was non-ionized when present in both the film and release medium. Hence, the loading of DIC was higher.

#### 3.8.2. Effect of Loading Method and Drug Physicochemical Properties on the Permeation Studies

The cumulative percentage of drug permeated from all studied post-loaded and in-situ loaded formulations are presented in Supplementary Figures S8 and S9, respectively. Based on the figures, the in vitro permeation profiles of PEO hydrogels were significantly affected by the loading method and physicochemical properties of drug. The permeation rates from post-loaded formulations are generally more rapid compared to the in situ loaded ones. This may be due to drug being deposited in microporous spaces of gel when post-loaded (as observed in SEM), instead of being embedded within polymeric network when loaded in situ. As a result, the post-loaded drugs were instantaneously released when hydrated. It is note-worthy that the EVA membrane was presoaked in phosphate buffer pH 7.2 before usage, to ensure all of its pores were hydrated. This may contribute to the near complete release of LID despite the high ionization, as the drug became solubilized when came into contact with buffer. The Co Tran 9702 EVA membrane was used to mimic skin permeability, as its pore size (50.8 μm) [64] is comparable to the pore size of normal abdominal skin (50 μm) [65]. In fact, the transdermal delivery of hydrophilic/ionized drugs is thought to be improved by hydrogels. The high water content of synthesized hydrogel films can act as natural penetration enhancer, as constant hydration of the stratum corneum through prolonged occlusion (24–48 h) achievable by using impermeable foil as backing layer of hydrogel films can lead to the swelling of corneocytes [66]. Besides, the polar water molecules may interact with the hydrophilic lipid heads, causing modification in the packing and orientation of lipid bilayer [67]. All of these are thought to create extra “pores” in the stratum corneum, allowing hydrophilic/ionized drug molecules to penetrate or permeate readily into the skin. Although the rod-shaped diclofenac acid crystals present in post-loaded DIC formulations were known to exhibit lower solubility compared to its sodium salt [68], the release of these crystal from the hydrogel formulation may be attributed to the ionization of the compound in phosphate buffer pH 7.2 (diclofenac acid p*K*a = 4.5) [69]. Hence, post-loaded DIC formulations at saturated concentration were capable of near complete drug release. Comparing between post-loaded formulations, drug flux (μg/cm^2^/h) of hydrophobic IBU is obviously different from those loaded with hydrophilic LID and DIC, as the drug took longer time (approximately 24 h) to reach near complete permeation. This suggested a different release mechanism for IBU from the hydrogel. It is thought that the release of hydrophilic/ionic compounds from hydrogels was governed by diffusion mechanism, whereas hydrophobic compounds were initially released by diffusion [70] and then through polymer degradation [71]. In addition, the formulation’s higher complex viscosity may contribute to the release retardation, as this indicated a more viscous gel layer, which can decrease the diffusion co-efficient of drug release in the hydrogels [72]. The mean total drug permeation from in situ loaded DIC formulations was only 63.04 (±2.18) % even after being left for longer than 72 h. This observation agreed with the suggestion of DIC interacting with PETRA under presence of UV radiation, as some DIC molecules were covalently bound to the polymer matrix.

For confirmation purposes, the total percentages of drug release obtained from the UV measurements were compared with the percentage release values calculated by weight method, using Equation (11). The results are tabulated in Table 8. From the table, it can be seen that percentage release value of post-loaded DIC hydrogel sample obtained from weight loss calculation were significantly higher than the ones measured by UV. This is due to the low crosslink density of formulations resulting significant loss of sample weight during the release study. For this reason, the percentage drug release for this studied formulation is more accurate when measured by UV. Apart from that, the total release percentages for all other formulations obtained from both methods agreed with one another.

#### 3.8.3. Effect of Loading Method and Drug Physicochemical Properties on the Release Kinetics

The in vitro permeation profiles of studied formulations were fit to different kinetic models and the results were tabulated in Table 9. The kinetic model best fitted to the permeation data was determined based on the regression coefficient (*R*^2^). It was found that post-loaded LID, DIC, and in situ loaded LID formulations followed both the zero order and Higuchi models. This dual release mechanism is verified by the Korsmeyer-Peppas model, as the obtained ‘*n*’ values indicated non-Fickian diffusion (0.5 < *n* < 1), which means that the overall release rate is contributed by both drug diffusion and polymer chain relaxation [3]. Although the permeation data from post-loaded IBU formulations were best fitted under zero order release model (constant drug release over time), their Korsmeyer-Peppas model ‘*n*’ value showed quasi-Fickian diffusion (*n* < 0.5), indicating a coupling of diffusion and erosion mechanism [73]. As for in situ loaded DIC formulations, the release rate followed first order kinetics, indicating the drug release rate is dependent on its concentration. Based on their corresponding Korsmeyer-Peppas ‘*n*’ value, the diffusion mechanism followed super case II transport mechanism (*n* > 1), indicated their release is most likely driven by macromolecular chain relaxation and swelling of hydrophilic polymer chain [74]. All these observations concluded that the release of drug from hydrogel films is heavily dependent on both drug physicochemical properties and material properties of hydrogel films.

## 4. Conclusions

The effects of loading methods and drug physicochemical properties on both the material and release properties of PEO hydrogel films were extensively studied. It was concluded that PEO hydrogel film patches synthesized via UV cross-linking can be formulated as transdermal patches with high drug loading, robust mechanical properties, and optimum drug release. The different loading methods resulted in significantly different gel profiles, and the suitability of each method is dependent on individual drug pharmaceutical properties and desired use; post-loaded films showed immediate drug release whereas in situ loaded films showed prolonged drug release. The majority of medicated PEO hydrogel formulations followed both zero order and Higuchi release pattern, which is highly desirable for controlled release formulations.

## Figures and Tables

**Table 1:** Physicochemical properties of model drugs.

Model drug	Molecular structure	Solubility in water	Log *P*	p*K*a	Melting point
LID	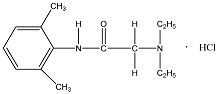	Water Soluble (50 mg/mL) [9]	Less than or equal to zero [10]	7.86 [11]	75–79 °C [9]
DIC	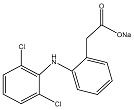	Water Soluble (20 mg/mL) ^a^	0.70 [12]	4.15 [13]	288–290 °C [14]
IBU	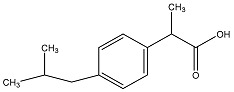	Slightly Soluble (21 mg/L at 25 °C) [15]	3.97 [15]	4.91 [15]	75–77.5 °C [15]

^a^ Experimental value.

**Table 2:** DSC temperature runs.

DSC heat/cool/heat temperature	Sample type
−80 °C/100 °C/−80 °C	Pure PEO, unmedicated PEO xerogel, LID, IBU, LID xerogels (post-loaded and in situ loaded), IBU xerogel (post-loaded)
−80 °C/350 °C/−80 °C	Unmedicated PEO xerogel, DIC, DIC xerogels (post-loaded and in situ loaded)

**Table 3:** Effect of drug physicochemical properties on the dependent variables involved in the swelling studies of in situ loaded PEO hydrogel films. Values in brackets indicate the standard deviation from the reported mean. (*n* = 4, * Significantly different from unmedicated hydrogel; *p* < 0.05).

PEO (*M*_w_ = 1000,000 g/mol) hydrogel cross-linked with 5% *w*/*w* PETRA									
Independent parameter	Unmedicated	LID (% *w*/*w*)				DIC (% *w*/*w*)			
		1.12	3.57	6.30	9.93	3.17	8.37	13.12	20.26
Dependent parameters									
Thickness of dry film (µm)	200	200	200	200	200	200	200	200	200
Thickness of swollen film (µm)	320	320	320	340	345	355	380	420	485
Equilibrium Swelling (%)	199.84 (±9.24)	203.37 (±2.34)	255.11 * (±9.22)	288.55 * (±6.46)	306.14 * (±8.79)	399.48 * (±9.94)	514.71 * (±19.43)	583.15 * (±41.51)	736.07 * (±84.93)
Gel Fraction (%)	85.84 (±1.65)	82.03 * (±0.31)	81.51 * (±0.98)	80.52 * (±1.01)	79.54 * (±0.42)	76.34 * (±1.72)	71.24 * (±4.56)	62.16 * (±5.81)	57.26 * (±7.34)
Average Molecular Weight Between Cross-links, ${\bar{M}}_{c}$ (g/mol)	734.48 (±62.36)	756.54 (±57.15)	1187.03 * (±84.17)	1508.77 * (±68.17)	1710.96 * (±136.21)	2921.48 * (±179.43)	4856.94 * (±363.97)	6243.55* (±886.04)	9927.18 * (±2424.43)
Cross-link Density, $\rho_{c}$ × 10^−4^ (mol/cm^3^)	16.45 (±1.56)	15.87 (±0.36)	10.16 * (±0.77)	7.92 * (±0.36)	7.05 * (±0.50)	4.12 * (±0.26)	2.48 * (±0.19)	1.96 * (±0.27)	1.26 * (±0.22)
Mesh Size, ξ (nm)	2.73 (±0.08)	2.76 (±0.04)	3.65 * (±0.06)	4.26 * (±0.12)	4.60 * (±0.13)	6.45 * (±0.25)	8.93 * (±0.43)	10.49 * (±0.96)	14.14 * (±2.12)

**Table 4:** Effect of loading method and drug physicochemical properties on the melting temperatures (*T*_m_) and degree of crystallinity of hydrogel polymeric film. *T*_m_ and corresponding $\Delta H_{f}$ values were obtained from second heating (cycle 3). Values in brackets indicate the standard deviation from reported mean (*n* = 3; * Significantly different from corresponding unmedicated hydrogels Significantly different within groups; *p <* 0.05).

Sample	Drug concentration (%*w*/*w*)	*T*_m_ (Cycle 3) (°C)	$\Delta H_{f}$ (J/g)	*X*_C_ (%)
Unmedicated	-	63.1 (±0.9)	103.7 (±3.4)	52.7 (±1.7)
Unmedicated (350 °C)	-	57.0 (±0.5)	145.5 (±1.9)	73.9 (±0.9)
Unmedicated (EtOH)	-	61.9 (±1.3)	97.7 (±0.9)	49.6 (±0.5)
Post-loaded LID	1.18	62.3 (±0.5)	103.0 (±4.5)	52.3 (±2.3)
	3.64	64.4 (±0.4)	95.2 (±3.3) *	48.4 (±1.7) *
	6.31	63.9 (±0.5)	90.3 (±1.4) *	45.9 (±0.7) *
	9.75	62.9 (±0.9)	87.3 (±2.1) *	44.4 (±1.1) *
Post-loaded DIC	3.28	58.5 (±0.2)	136.6 (±2.4) *	69.4 (±1.2) *
	8.39	56.4 (±0.2) *	115.8 (±1.9) *	58.8 (±0.9) *
	13.62	55.6 (±0.2) *	112.1 (±4.0) *	56.9 (±2.1) *
	20.68	54.3 (±0.3) *	105.9 (±2.3) *	53.8 (±1.2) *
Post-loaded IBU	15.44	60.2 (±0.2)	75.6 (±2.7) *	38.4 (±1.4) *
	31.01	55.3 (±0.1) *	61.3 (±3.0) *	31.1 (±1.5) *
	45.48	51.3 (±2.1) *	49.8 (±5.0) *	25.3 (±2.5) *
	59.09	51.0 (±5.1) *	46.7 (±5.9) *	23.8 (±3.0) *
In situ Loaded LID	1.12	64.6 (±0.8)	100.1 (±3.7)	50.9 (±1.9)
	3.57	64.6 (±0.5)	102.3 (±3.8)	52.3 (±1.9)
	6.3	64.5 (±0.2)	103.2 (±0.3)	52.4 (±0.1)
	9.93	64.2 (±0.5)	98.9 (±1.1)	50.2 (±0.6)
In situ Loaded DIC	3.17	57.0 (±0.5)	139.0 (±7.8)	70.6(±3.9)
	8.37	56.0 (±1.8)	133.7 (±5.4) *	68.0 (±2.7) *
	13.12	55.4 (±0.5) *	119.0 (±0.2) *	60.5 (±1.0) *
	20.26	52.6 (±0.4) *	99.8 (±2.6) *	50.7 (±1.3) *

**Table 5:** Effect of loading method and drug physicochemical properties on the mechanical properties. Values in brackets indicate the standard deviation from reported mean (*n* = 3; * Significantly different from corresponding unmedicated hydrogels; *p <* 0.05).

Sample	Drug concentration (% *w*/*w*)	Tensile strength (MPa)	Percentage elongation (%)	Young’s modulus (MPa)
Unmedicated	-	1.25 (±0.05)	19.84 (±2.75)	7.87 (±0.30)
Unmedicated (EtOH)	-	1.21 (±0.13)	19.86 (±2.94)	7.61 (±0.11)
Post-loaded LID	1.18	1.20 (±0.09)	20.23 (±2.03)	7.41 (±0.53)
	3.64	1.25 (±0.08)	20.05 (±2.42)	7.79 (±0.58)
	6.31	1.31 (±0.11)	21.75 (±1.79)	7.52 (±0.46)
	9.75	1.32 (±0.07)	22.64 (±1.44)	7.28 (±0.47)
Post-loaded DIC	3.28	1.20 (±0.07)	20.49 (±1.09)	7.31 (±0.51)
	8.39	1.15 (±0.11)	21.27 (±1.89)	6.75 (±0.59) *
	13.62	1.12 (±0.10)	22.39 (±2.36)	6.24 (±0.42) *
	20.68	1.08 (±0.11) *	22.74 (±1.96)	5.93 (±0.21) *
Post-loaded IBU	15.44	1.20 (±0.08)	20.12 (±1.01)	7.45 (±0.48)
	31.01	1.19 (±0.05)	21.87 (±2.10)	6.80 (±0.55) *
	45.48	1.17 (±0.05)	22.36 (±2.42)	6.54 (±0.57) *
	59.09	1.15 (±0.03)	23.95 (±2.97)	6.00 (±0.71) *
In situ Loaded LID	1.12	1.14 (± 0.14)	22.18 (±2.61)	6.42 (±0.24) *
	3.57	0.82 (±0.02) *	26.37 (±2.70) *	3.88 (±0.16) *
	6.3	0.56 (±0.03) *	32.53 (±3.28) *	2.15 (±0.25) *
	9.93	0.50 (±0.04) *	40.01 (±3.05) *	1.56 (±0.18) *
In situ Loaded DIC	3.17	0.19 (±0.03) *	24.13 (±3.49) *	0.98 (±0.04) *
	8.37	0.14 (±0.02) *	35.30 (±5.60) *	0.50 (±0.03) *
	13.12	0.10 (±0.02) *	43.39 (±8.39) *	0.29 (±0.04) *
	20.26	0.05 (±0.02) *	54.68 (±7.80) *	0.11 (±0.04) *

**Table 6:** Effect of loading method and drug physicochemical properties on the mechanical properties. Values in brackets indicate the standard deviation from reported mean (*n* = 3; * Significantly different from corresponding unmedicated hydrogels; *p <* 0.05).

Sample	Drug concentration (% *w*/*w*)	Elastic modulus, *G*’ 10^4^ (Pa)	Viscous modulus, *G*” 10^4^ (Pa)	Critical strain ($\gamma_{0}$)	Complex viscosity at frequency = 10 Hz, η* 10^3^ (Pa·s)
Unmedicated	-	9.27 (±0.12)	1.04 (±0.33)	1.68	1.57 (±0.21)
Unmedicated (EtOH)	-	9.03 (±0.10)	1.14 (±0.29)	1.58	1.65 (±0.18)
Post-loaded LID	1.18	9.40 (±0.14)	1.05 (±0.08)	1.59	1.57 (±0.05)
	3.64	10.52 (±0.19) *	1.15 (±0.15)	0.99	1.67 (±0.17)
	6.31	10.71 (±0.16) *	1.18 (±0.07)	0.67	1.69 (±0.08)
	9.75	11.54 (±0.22) *	1.26 (±0.07)	0.4	1.78 (±0.12)
Post-loaded DIC	3.28	9.89 (±0.22) *	1.11 (±0.29)	1.58	1.63 (±0.12)
	8.39	11.79 (±0.03) *	1.33 (±0.33)	1.25	1.83 (±0.06)
	13.62	14.44 (±0.23) *	1.41 (±0.30)	0.84	1.97 (±0.25)
	20.68	17.16 (±0.21) *	1.64 (±0.27) *	0.53	2.06 (±0.29)
Post-loaded IBU	15.44	10.97 (±0.38) *	1.16 (±0.41)	1.26	1.69 (±0.16)
	31.01	12.18 (±0.45) *	1.87 (±0.17) *	1	2.25 (±0.33) *
	45.48	17.65 (±0.71) *	3.12 (±0.42) *	0.62	3.02 (±0.45) *
	59.09	20.54 (±0.48) *	4.32 (±0.19) *	0.31	3.69 (±0.31) *
In situ Loaded LID	1.12	10.20 (± 0.13) *	1.13 (±0.33)	0.79	1.64 (±0.04)
	3.57	24.35 (±0.23) *	1.54 (±0.20)	0.63	3.58 (±0.09) *
	6.3	28.62 (±0.30) *	1.59 (±0.27)	0.63	4.87 (±0.15) *
	9.93	39.22 (±0.14) *	1.75 (±0.19) *	0.5	6.34 (±0.34) *
In situ Loaded DIC	3.17	0.42 (±0.13) *	0.03 (±0.01) *	0.63	0.04 (±0.01) *
	8.37	-	-	-	-
	13.12	-	-	-	-
	20.26	-	-	-	-

**Table 7:** Effect of loading method and drug physicochemical properties on the drug ionization. Values in brackets indicate the standard deviation from reported mean (*n* = 3; * Significantly different from corresponding unmedicated hydrogels; *p <* 0.05).

Sample	Drug concentration (% *w*/*w*)	Apparent pH	Drug ionization in film (%)	Drug ionization in phosphate buffer pH 7.2 (%)
Unmedicated	-	5.61 (±0.01)	-	-
Unmedicated (EtOH)	-	5.61 (±0.00)	-	-
Post-loaded LID	9.75	5.80 (±0.00) *	99.14	82.05
Post-loaded DIC	20.68	6.71 (±0.00) *	0.27	0.09
Post-loaded IBU	59.09	4.66 (±0.01) *	-	-
In situ Loaded LID	9.93	6.34 (±0.01) *	97.07	82.05
In situ Loaded DIC	20.26	6.75 (±0.01) *	0.25	0.09

**Table 8:** Comparison of total percentage drug release obtained from release studies to percentage release values calculated by weight measurements. Values in brackets indicate the standard deviation from reported mean (*n* = 4; * Significantly different between compared values).

Formulation	Drug concentration (% *w*/*w*)	Mean total percentage drug release from UV measurements (%)	Mean total percentage drug release based on weight measurements (%)
Post-loaded LID	9.75	92.60 (±2.26)	93.77 (±0.24)
Post-loaded DIC	20.68	93.90 (±2.27)	94.52 (±0.59)
Post-loaded IBU	59.09	96.82 (±2.95)	99.60 (±0.62)
In situ Loaded LID	9.93	97.53 (±1.08)	98.45 (±0.12)
In situ Loaded DIC	20.26	63.04 (±2.18) *	120.75 (±2.32) *

**Table 9:** Effect of loading method and drug physicochemical properties on the release kinetics.

Formulation	Drug concentration (% *w*/*w*)	Zero order		First order		Higuchi model		Korsmeyer-peppas model		
		*R*^2^	*K*_0_	*R*^2^	*K*_1_	*R*^2^	*K*_2_	*R*^2^	*K*_3_	*n*
**Post-loaded LID**	9.75	0.961	13.016	0.962	1.511	0.961	13.016	0.999	1.536	0.788
**Post-loaded DIC**	20.68	0.958	15.349	0.966	1.595	0.958	15.349	0.995	1.621	0.733
**Post-loaded IBU**	59.09	0.993	3.343	0.986	0.575	0.995	4.571	0.989	0.006	0.015
**In situ Loaded LID**	9.93	0.994	8.099	0.987	0.744	0.994	8.099	0.970	0.908	0.786
**In situ Loaded DIC**	20.26	0.956	6.3498	0.982	1.398	0.962	6.774	0.981	1.403	1.811

## Supplementary Materials

The following are available online at www.mdpi.com/2073-4360/9/7/286/s1, Figure S1: DSC thermograms of hydrogel formulations containing the highest load for (a) LID, (b) DIC, and (c) IBU in comparison to unmedicated ones and corresponding pure drug, Figure S2: DSC thermograms of hydrogel formulations containing the highest load for (a) LID, (b) DIC, and (c) IBU in comparison to unmedicated ones and corresponding pure drug, Figure S3: FT-IR spectra of post-loaded LID formulations in comparison to pure LID and unmedicated PEO hydrogel film, Figure S4: FT-IR spectra of post-loaded DIC formulations in comparison to pure DIC and unmedicated PEO xerogel film, Figure S5: FT-IR spectra of post-loaded IBU formulations in comparison to pure IBU and unmedicated PEO xerogel film, Figure S6: FT-IR spectra of in-situ loaded LID formulations in comparison to pure LID and unmedicated PEO xerogel film, Figure S7: FT-IR spectra of in-situ loaded DIC formulations in comparison to pure DIC and unmedicated PEO xerogel film, Figure S8: Permeation of post-loaded model drugs from hydrogels in pH 7.2 phosphate buffer through EVA membrane, Figure S9: Permeation of in-situ loaded model drugs from hydrogels in pH 7.2 phosphate buffer through EVA membrane.
