# Supplementary material for: Effect of Drug Loading Method and Drug Physicochemical Properties on the Material and Drug Release Properties of Poly (Ethylene Oxide) Hydrogels for Transdermal Delivery
Source: Polymers (Basel). 2017 Jul 19;9(7):286. doi: 10.3390/polym9070286 (PMC6432290; doi:10.3390/polym9070286)
Supplement: Supplementary file 1 [file polymers-09-00286-s001.pdf]

Supplementary Figures

Effect of drug loading method and drug physicochemical properties on the material and drug release properties on the material and drug release properties of poly (ethylene oxide) hydrogels for transdermal delivery

Rachel Shet Hui Wong and Kalliopi Dodou*

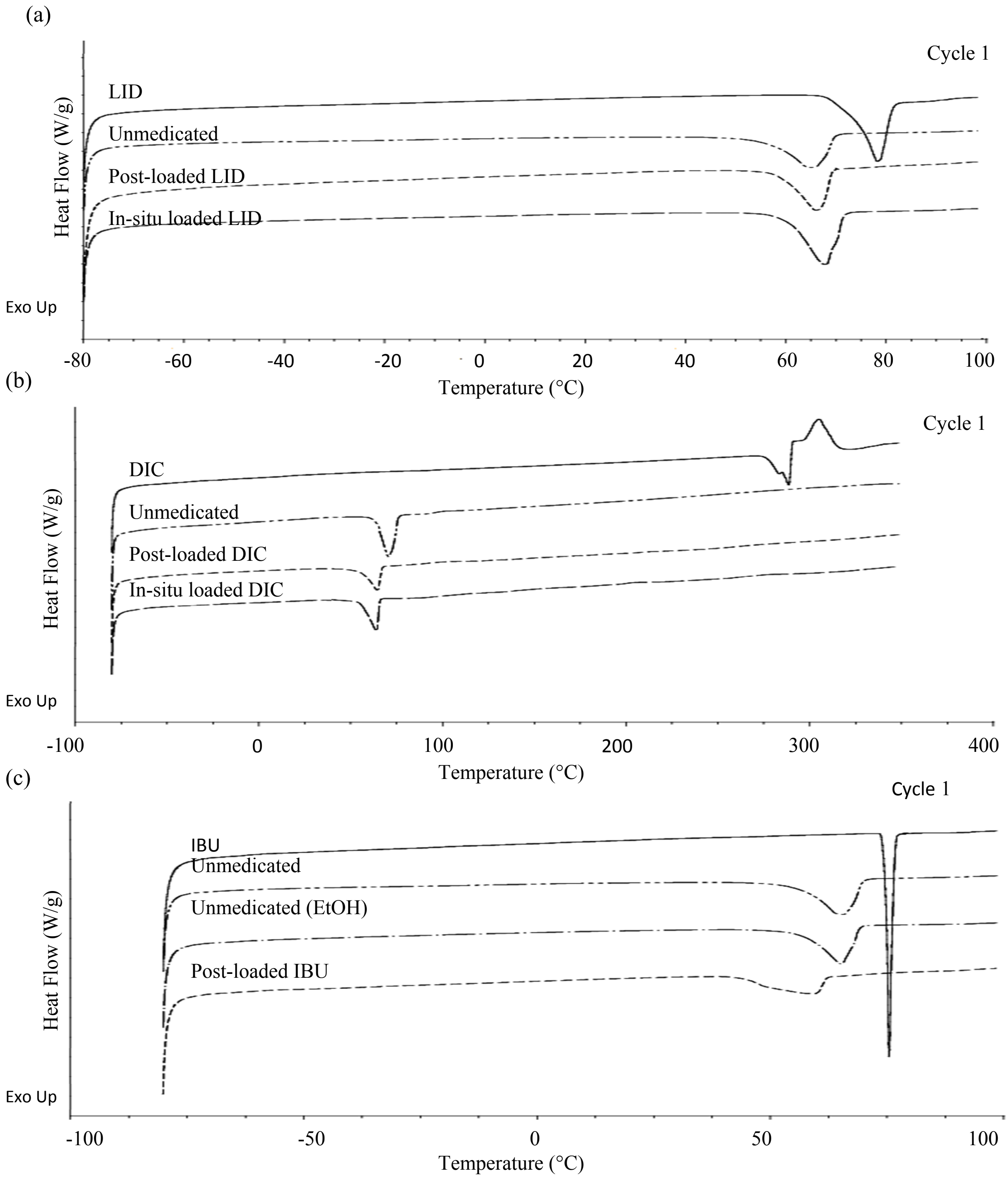

Figure S1. DSC thermograms of hydrogel formulations containing the highest load for (a) LID, (b) DIC, and (c) IBU in comparison to unmedicated ones and corresponding pure drug. Thermograms were obtained from first heating (cycle 1).

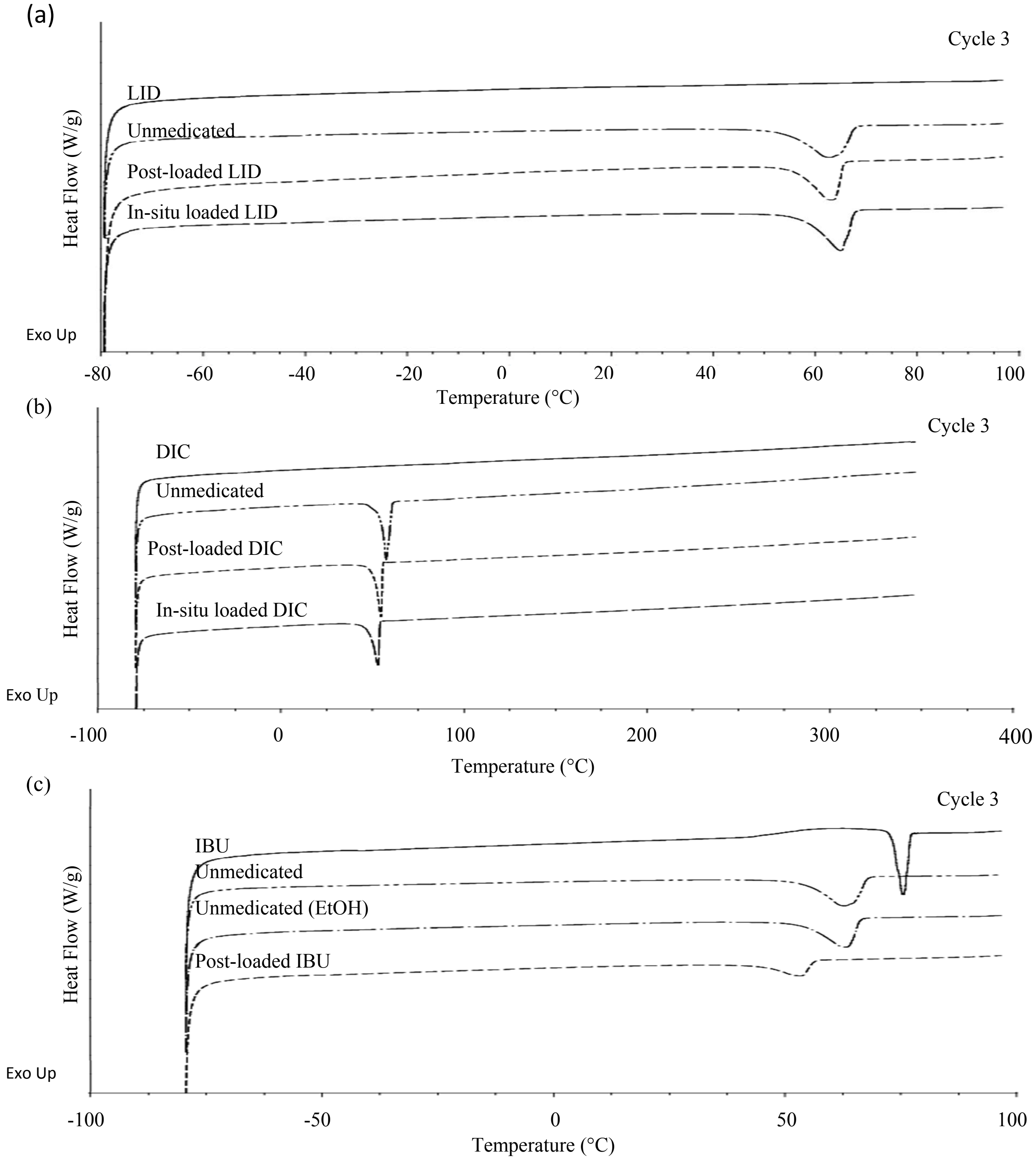

Figure S2. DSC thermograms of hydrogel formulations containing the highest load for (a) LID, (b) DIC, and (c) IBU in comparison to unmedicated ones and corresponding pure drug. Thermograms were obtained from second heating (cycle 3).

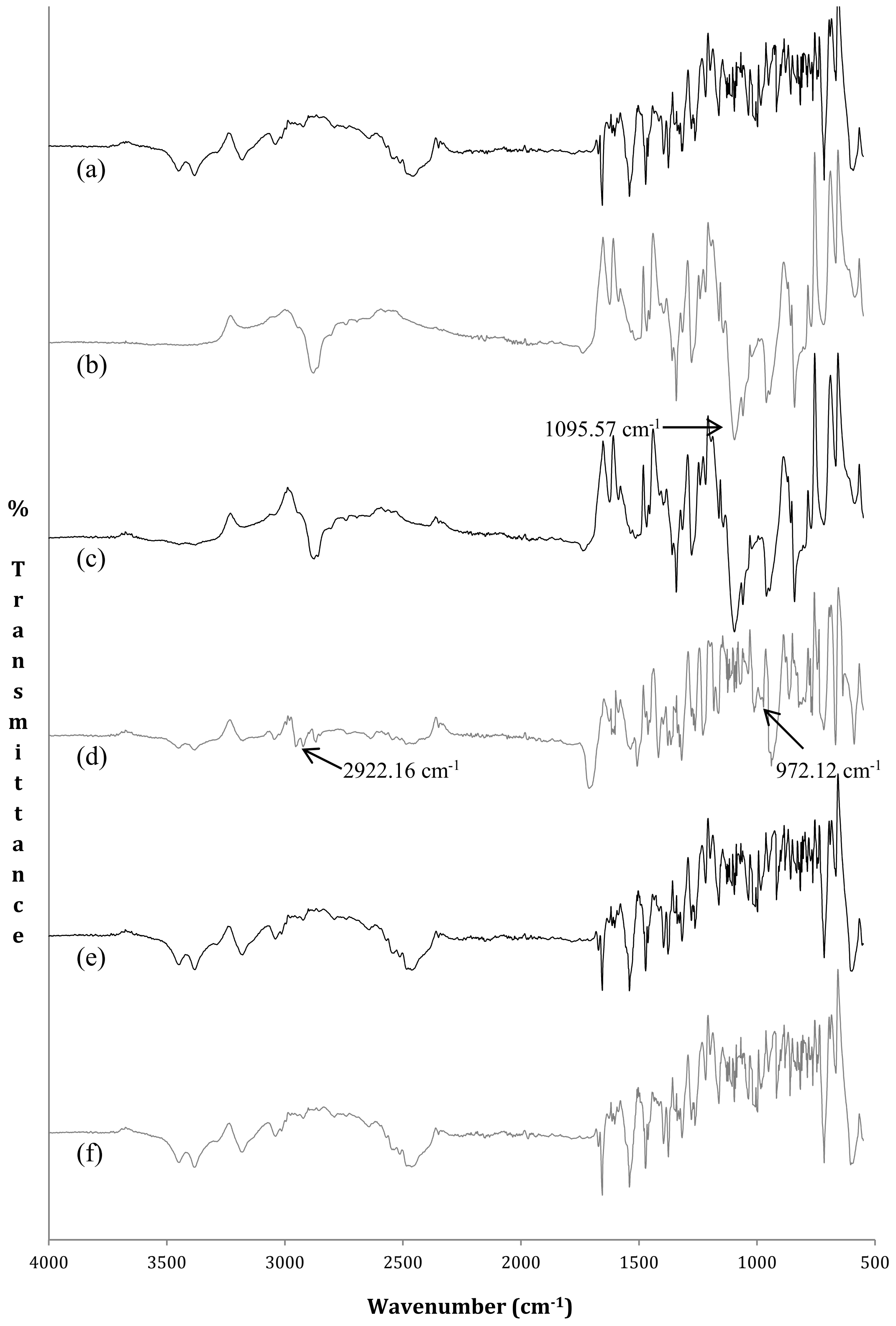

Figure S3. FT-IR spectra of post-loaded LID formulations in comparison to pure LID and unmedicated PEO hydrogel film. (a) Pure LID, (b) unmedicated PEO xerogel film, (c) PEO-LID 1.12% w/w (25% saturation), (d) PEO-LID 3.64% w/w (50% saturation), (e) PEO-LID 6.31% w/w (75% saturation), (f) PEO-LID 9.75% w/w (100% saturation).

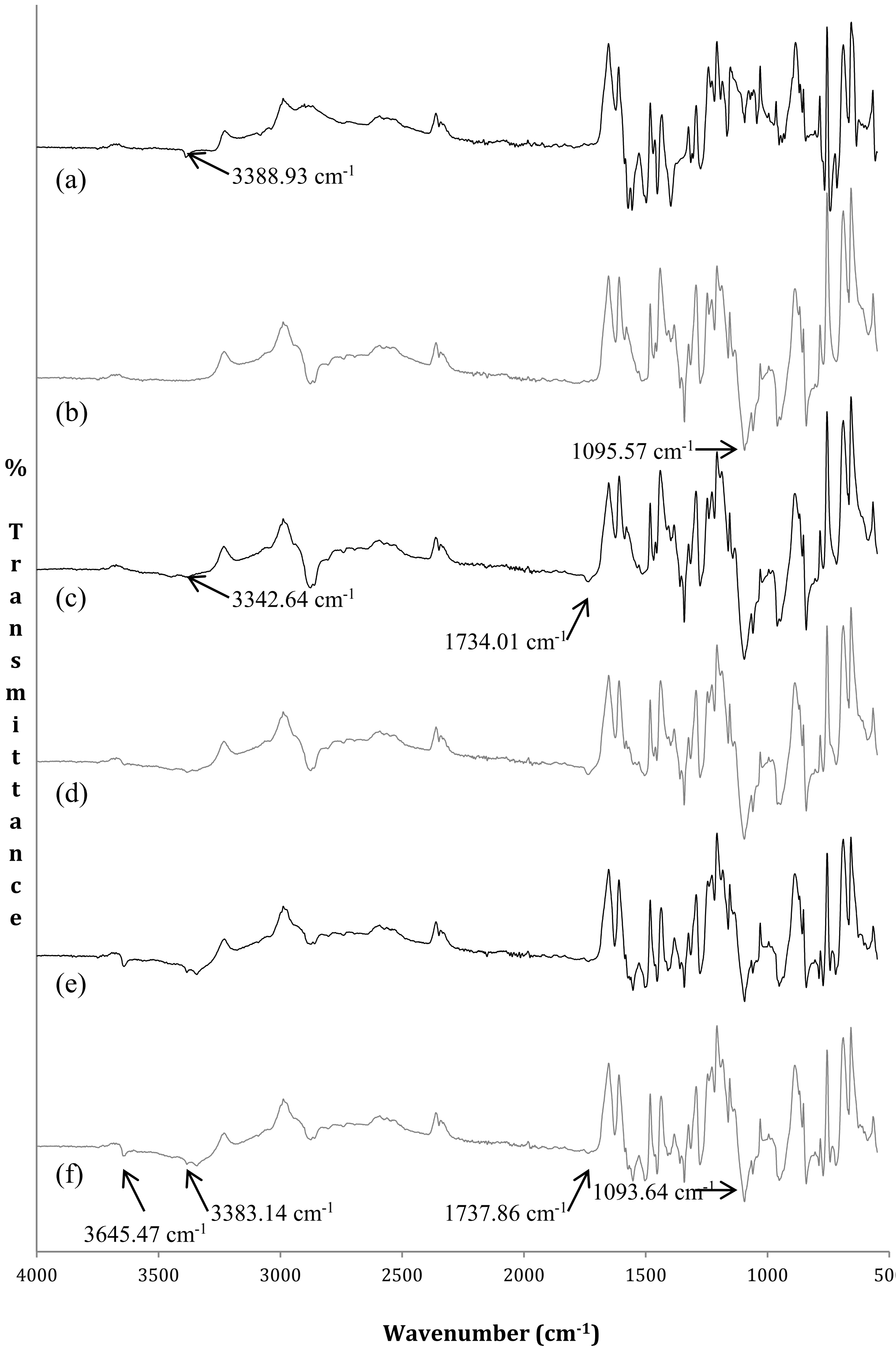

Figure S4. FT-IR spectra of post-loaded DIC formulations in comparison to pure DIC and unmedicated PEO xerogel film. (a) Pure DIC, (b) unmedicated PEO xerogel film, (c) PEO-DIC 3.28% w/w (25% saturation), (d) PEO-DIC 8.39% w/w (50% saturation), (e) PEO-DIC 13.62% w/w (75% saturation), (f) PEO-DIC 20.68% w/w (100% saturation).

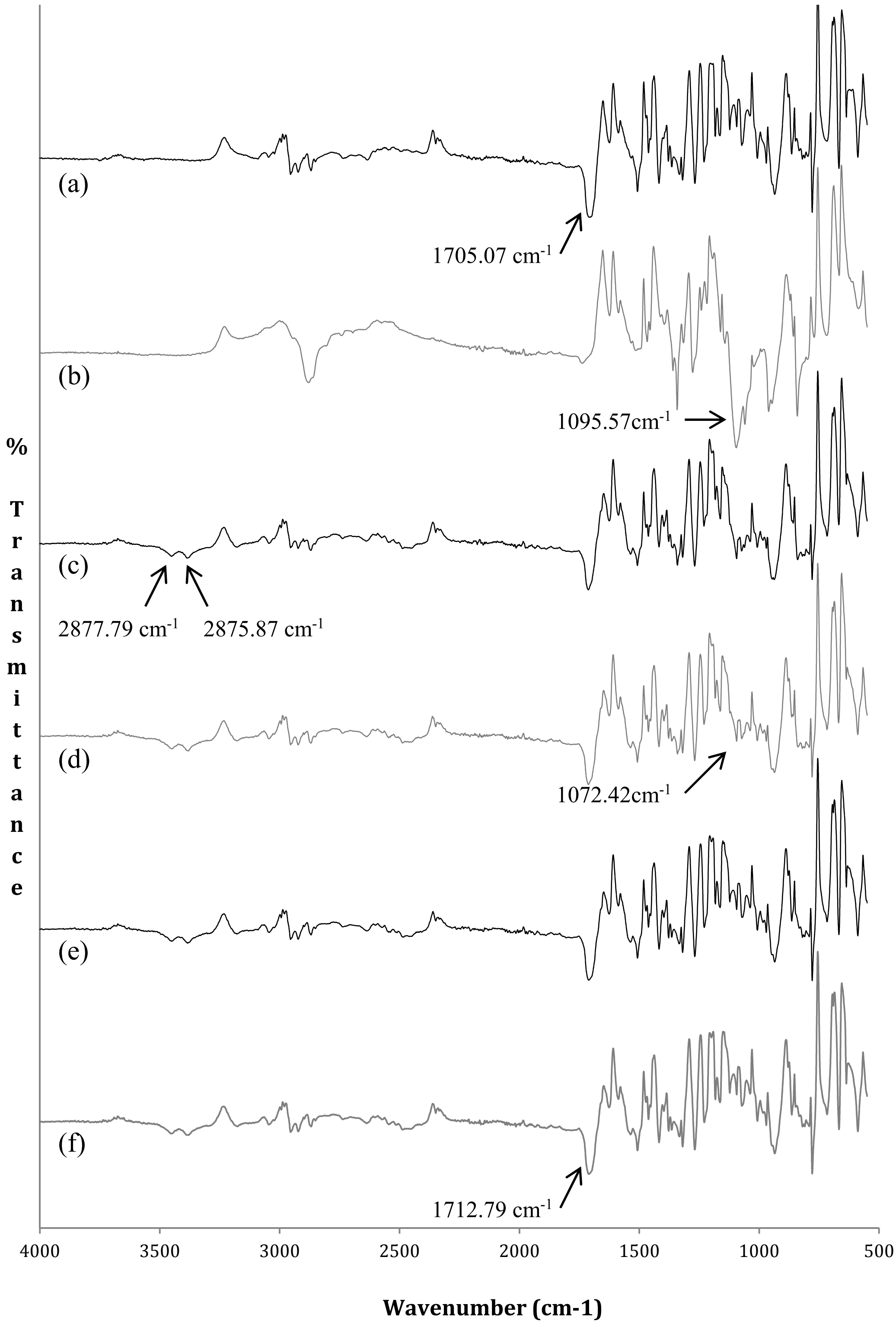

Figure S5. FT-IR spectra of post-loaded IBU formulations in comparison to pure IBU and unmedicated PEO xerogel film. (a) Pure IBU, (b) unmedicated PEO xerogel film, (c) PEO-IBU 15.54% w/w (25% saturation), (d) PEO-IBU 31.01% w/w (50% saturation), (e) PEO-IBU 45.48% w/w (75% saturation), (f) PEO-IBU 59.09% w/w (100% saturation).

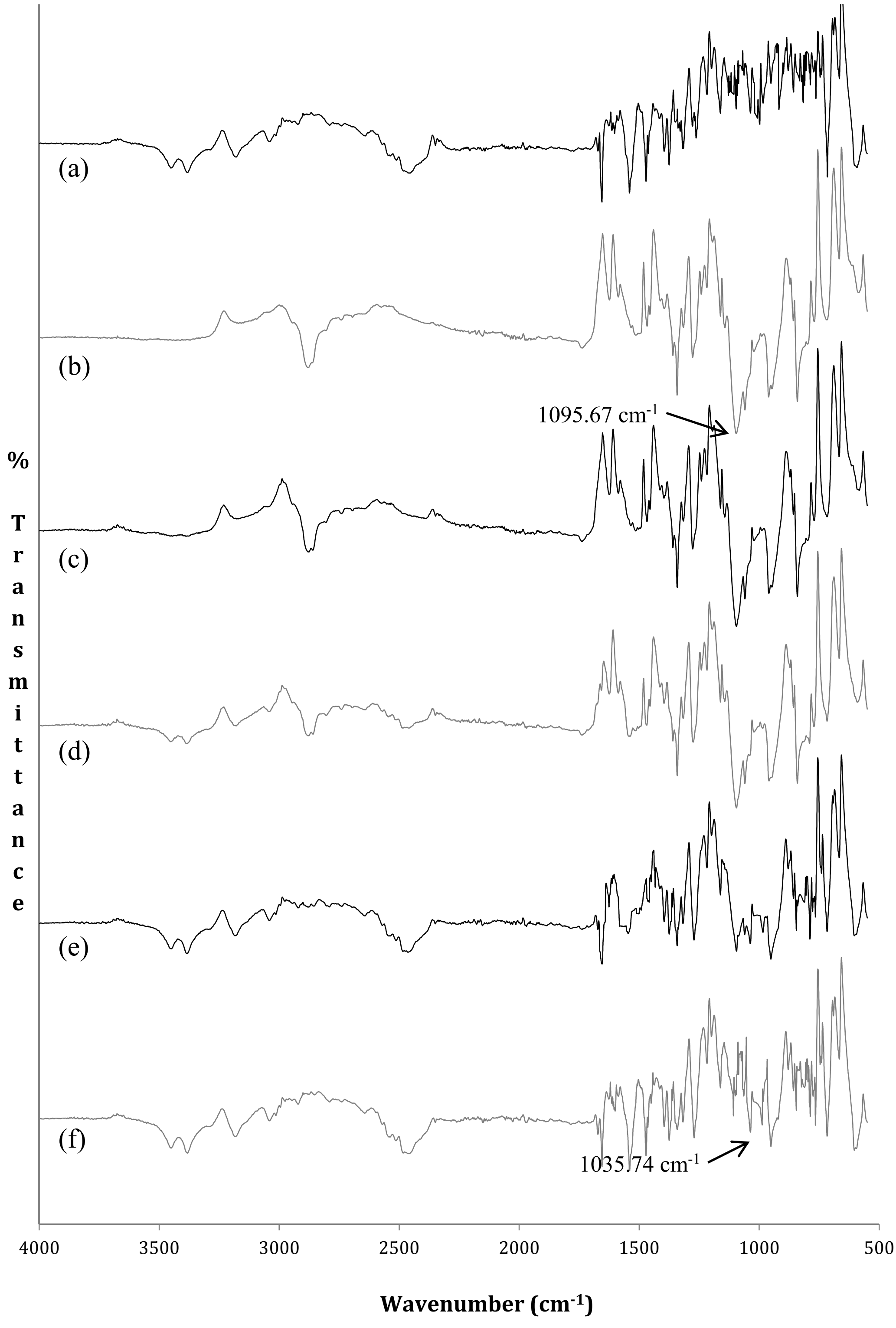

Figure S6. FT-IR spectra of in-situ loaded LID formulations in comparison to pure LID and unmedicated PEO xerogel film. (a) Pure LID, (b) unmedicated PEO xerogel film, (c) PEO-LID 1.12% w/w, (d) PEO-LID 3.57% w/w, (e) PEO-LID 6.30% w/w, (f) PEO-LID 9.93% w/w.

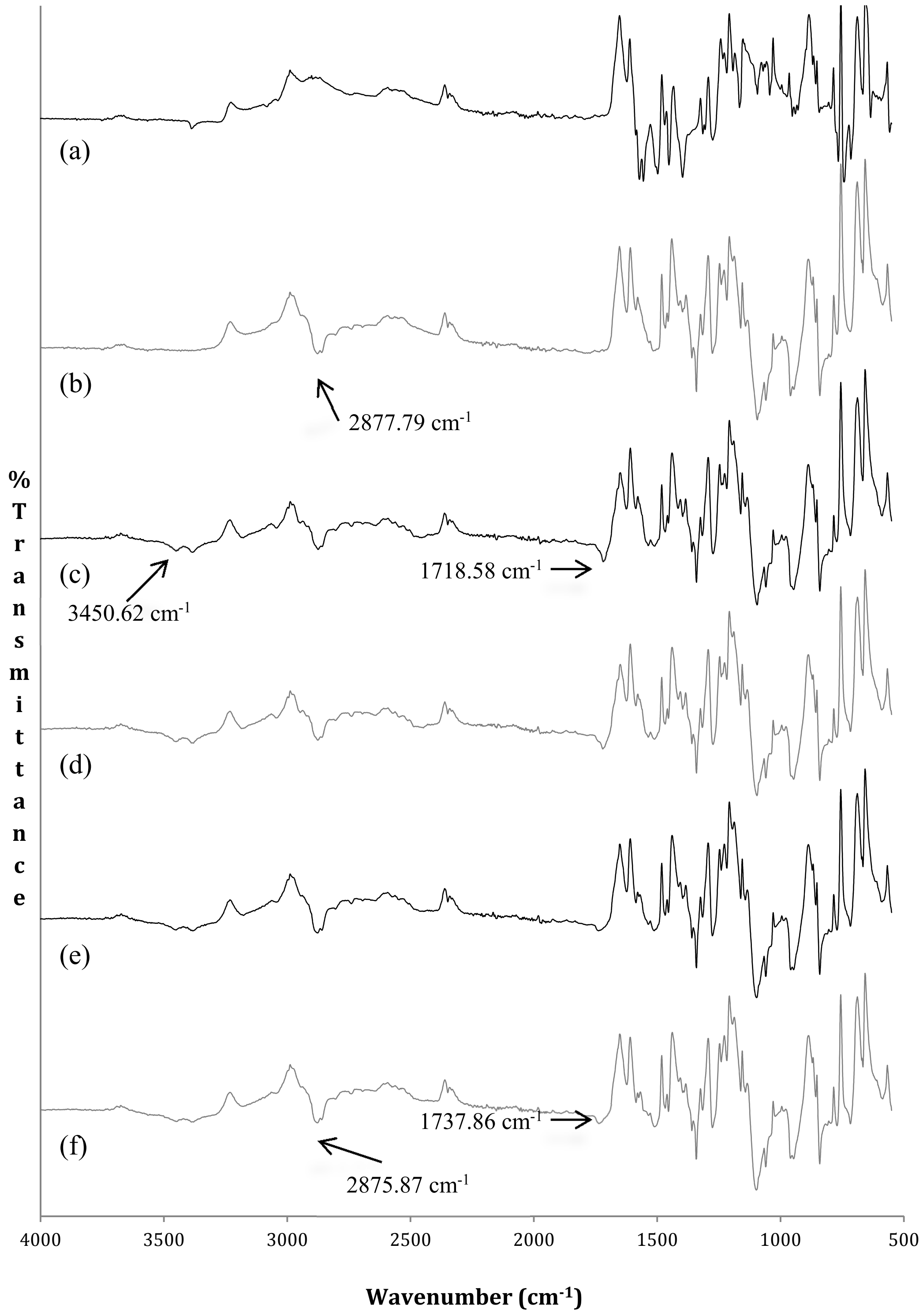

Figure S7. FT-IR spectra of in-situ loaded DIC formulations in comparison to pure DIC and unmedicated PEO xerogel film. (a) Pure DIC, (b) unmedicated PEO xerogel film, (c) PEO-DIC 3.17% w/w, (d) PEO-DIC 8.37% w/w, (e) PEO-DIC 13.12% w/w, (f) PEO-DIC 20.26% w/w.

A)

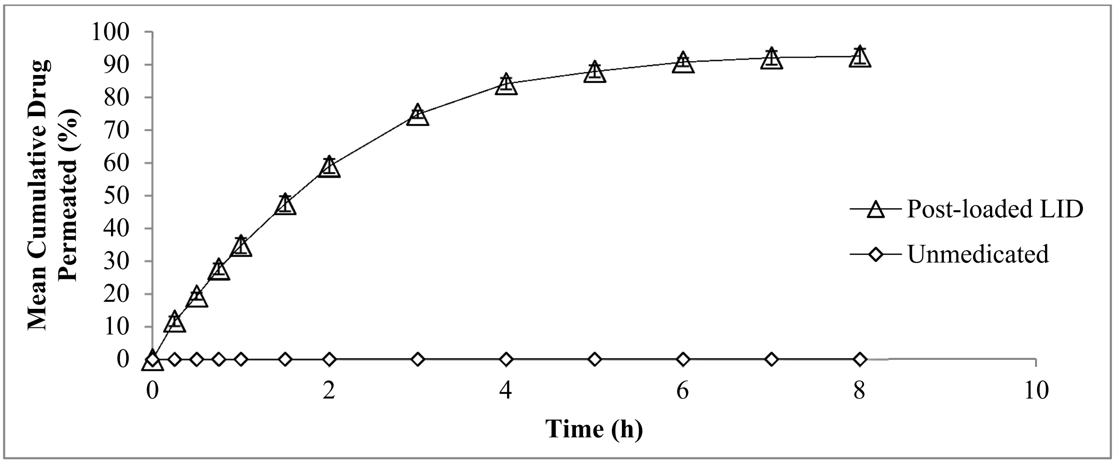

B)

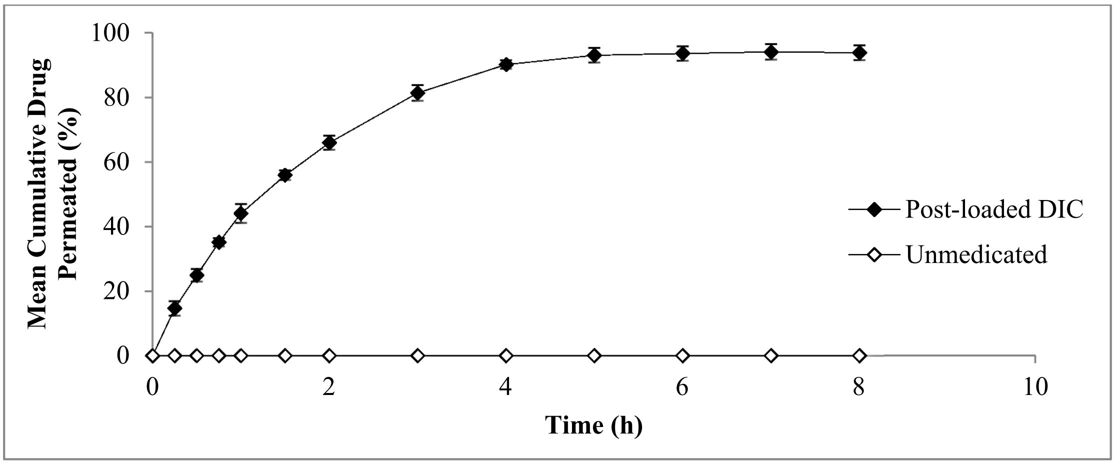

C)

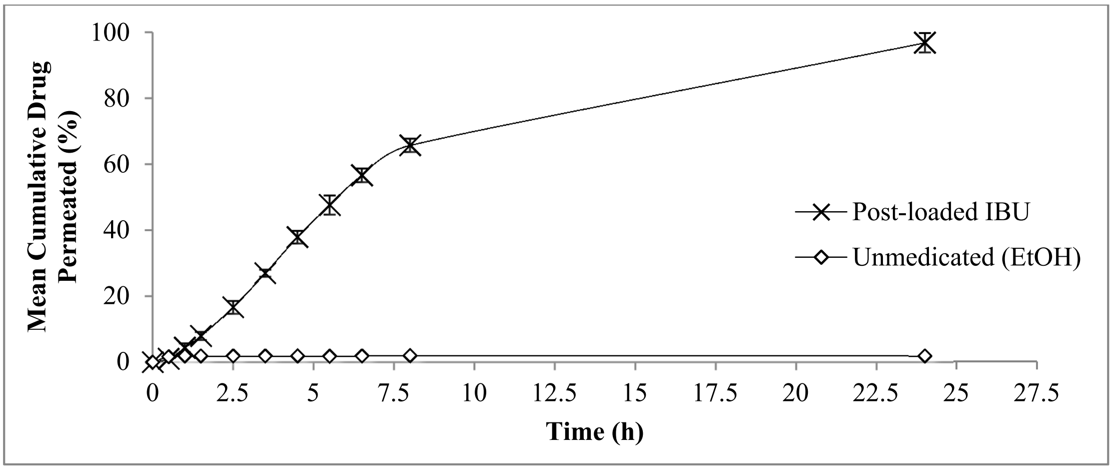

Figure S8. Permeation of post-loaded model drugs from hydrogels in pH 7.2 phosphate buffer through EVA membrane. A) LID, B) DIC, C) IBU.

A)

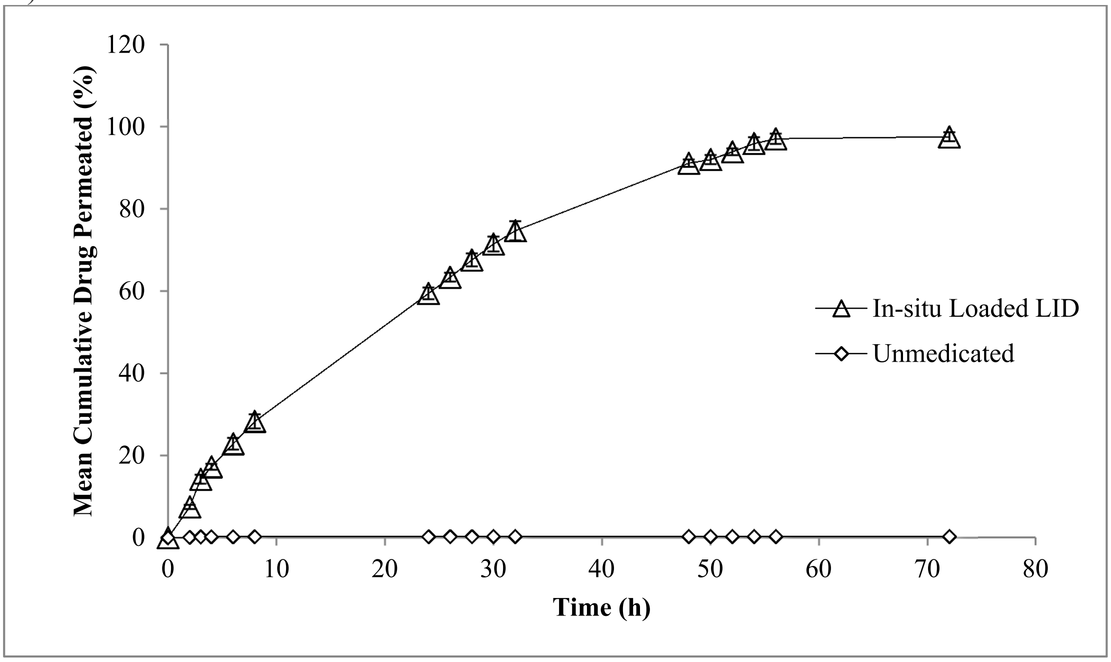

B)

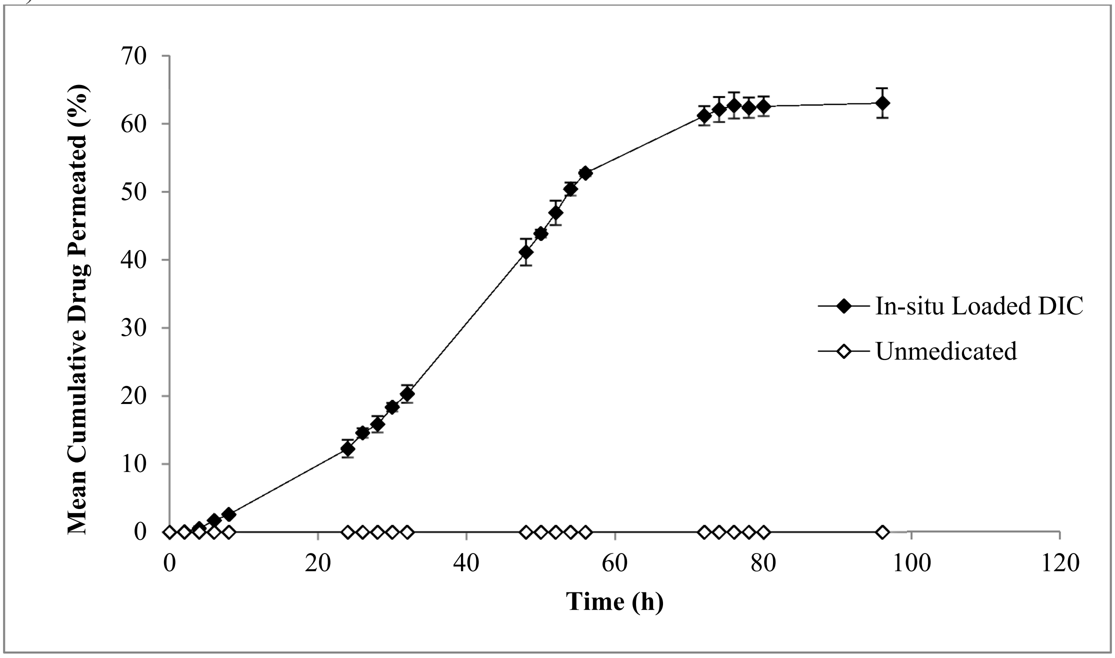

Figure S9. Permeation of in-situ loaded model drugs from hydrogels in pH 7.2 phosphate buffer through EVA membrane. A) LID, B) DIC.
